# Efficient, Long Term Production of Monocyte-Derived Macrophages from Human Pluripotent Stem Cells under Partly-Defined and Fully-Defined Conditions

**DOI:** 10.1371/journal.pone.0071098

**Published:** 2013-08-12

**Authors:** Bonnie van Wilgenburg, Cathy Browne, Jane Vowles, Sally A. Cowley

**Affiliations:** Sir William Dunn School of Pathology, University of Oxford, Oxford, United Kingdom; University of Sao Paulo - USP, Brazil

## Abstract

Human macrophages are specialised hosts for HIV-1, dengue virus, *Leishmania* and *Mycobacterium tuberculosis*. Yet macrophage research is hampered by lack of appropriate cell models for modelling infection by these human pathogens, because available myeloid cell lines are, by definition, not terminally differentiated like tissue macrophages. We describe here a method for deriving monocytes and macrophages from human Pluripotent Stem Cells which improves on previously published protocols in that it uses entirely defined, feeder- and serum-free culture conditions and produces very consistent, pure, high yields across both human Embryonic Stem Cell (hESC) and multiple human induced Pluripotent Stem Cell (hiPSC) lines over time periods of up to one year. Cumulatively, up to ∼3×10^7^ monocytes can be harvested per 6-well plate. The monocytes produced are most closely similar to the major blood monocyte (CD14^+^, CD16^low^, CD163^+^). Differentiation with M-CSF produces macrophages that are highly phagocytic, HIV-1-infectable, and upon activation produce a pro-inflammatory cytokine profile similar to blood monocyte-derived macrophages. Macrophages are notoriously hard to genetically manipulate, as they recognise foreign nucleic acids; the lentivector system described here overcomes this, as pluripotent stem cells can be relatively simply genetically manipulated for efficient transgene expression in the differentiated cells, surmounting issues of transgene silencing. Overall, the method we describe here is an efficient, effective, scalable system for the reproducible production and genetic modification of human macrophages, facilitating the interrogation of human macrophage biology.

## Introduction

Macrophages are widely distributed throughout the tissues of the body, being involved in tissue repair and homeostasis, as well as being a key component of the immune system. They are hosts for various macrophage-tropic pathogens, including HIV-1, dengue virus, *Leishmania* and *Mycobacterium tuberculosis*. However, macrophage research has been hampered by the difficulty of obtaining and working with this terminally differentiated cell-type. Blood monocyte-derived macrophages have been used for many years as a relevant experimental model of human *in vivo* macrophages. However, relatively large amounts of blood are usually required from each donor. Each donor’s cells will be in significantly different physiological states on different occasions and each donor is genetically different. It is necessary, therefore, to use many different donors on multiple occasions, to ensure that an experimental observation is representative. An additional issue is that macrophages are particularly refractory to genetic manipulation, yet only by manipulating the expression of specific genes in an otherwise fixed genetic background can the role of those gene products be properly studied. Animal models do not adequately overcome these problems as species differences can mean results are not always relevant to human immunopathology. Meanwhile, human myeloid cell lines, such as THP-1, are karyotypically abnormal and, by definition, are not terminally differentiated like macrophages. CD34^+^ haematopoietic stem cells isolated from cord blood or bone marrow offer a tractable system for genetic modification. However, unlike hiPSC and hESC, collectively referred to as Pluripotent Stem Cells (PSC), CD34^+^ haematopoietic stem cells are challenging to expand ex-vivo and do not self-renew [1]. Although CD34^+^ haematopoietic stem cells could be characterized, they are mostly used without characterization of their patient background, whereas many PSC lines have been extensively characterized [2]. Therefore, pluripotent stem cell-derived macrophages offer an attractive alternative system for deriving terminally differentiated, karyotypically normal, genetically consistent human macrophages.

Several groups have published methods for producing monocytes/and macrophages from PSC [3], [4], [5], [6], [7]. However, these methods are technically complicated, because they involve coculture on mouse stromal cells (e.g. OP9 cells), and/or purification of progenitor cells from partially-differentiated cultures prior to differentiation to monocytes. Moreover, none of these protocols are amenable to scaling, and none uses fully defined culture conditions.

We have previously described a simpler method for producing functional monocytes and macrophages from hESC [8]. This method utilises the spontaneous differentiation of hESC into embryoid bodies (comprising ectoderm, mesoderm and endoderm), followed by directed differentiation along the myeloid lineage by IL-3 and M-CSF, to produce a homogeneous population of monocytes (esMC), which can be further differentiated into macrophages (esMDM). These macrophages are phenotypically and functionally comparable to blood monocyte-derived macrophages (bMDM). However, although this method can yield over 1×10^7^ monocytes (MC) from a 6-well plate of differentiation cultures [8], such high yields are generally only achieved once a week, for 1–3 weeks, after which yields dramatically decrease. Moreover, there is substantial variability in monocyte yield across different hESC and hiPSC lines. Serum is the most likely component of the medium to cause the tail-off in productivity and the variability between differentiation runs, as it likely contains many growth factors, not all of which are necessarily optimal for myeloid lineage maintenance. Therefore, to optimize differentiation, we sought to remove serum during monocytopoiesis. We have also developed a fully chemically defined, serum- and feeder-free protocol for monocyte and macrophage production, which substantially improves reproducibility. Using this serum-free protocol we can now generate differentiation cultures which continue to produce harvestable, uniform monocytes for many months and even up to 1 year. We demonstrate that this method also works for hiPSC. We also show that this differentiation system supports the expression of transgenes in hES-derived monocytes/and macrophages following delivery in lentiviral vectors at the stem cell level.

## Results

### Efficient, Long-term Production of PSC-MDM Using Defined Media

**Figure 1:**
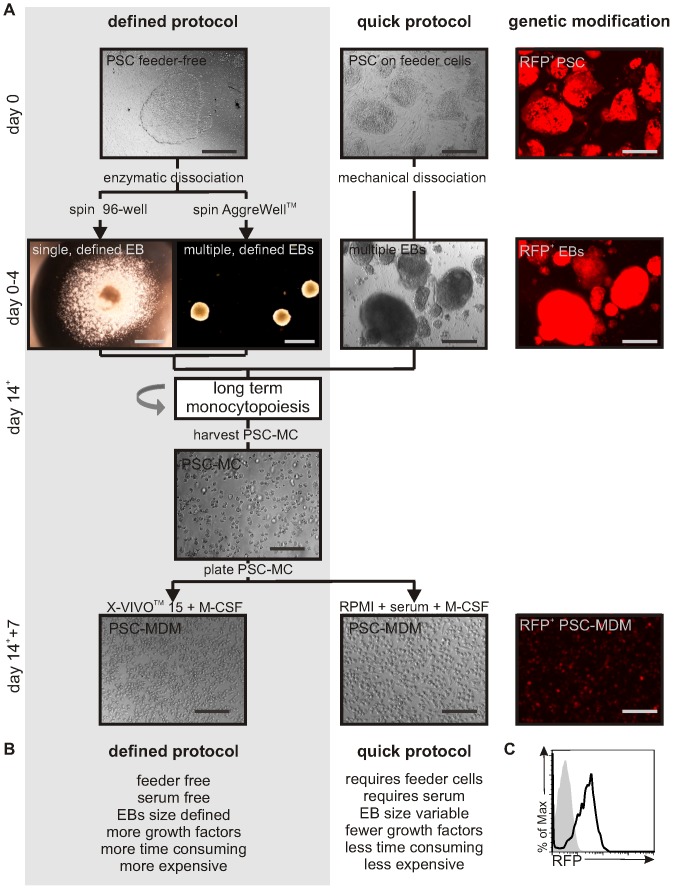
Protocols for the long-term production of PSC-MDM which are genetically modifiable. A) Diagram outlining the defined and quick protocols for the differentiation of MDM from PSC. Using the defined protocol, PSC colonies are cultured on feeder-free Synthemax™ tissue culture plates and uniform EBs are generated using centrifugation of PSC in a 96-well plate (to isolate single EBs) or in a AggreWell™ plate. The defined EBs are formed in mTeSR™-1 medium and require the addition of three growth factors. Using the quick protocol, PSC colonies are cultured on inactivated feeder cells and EBs are generated using mechanical dissociation and culture in PSC media in ultra-low adherence plates. After 4 days of EB culture, EBs are transferred to regular tissue culture plates and differentiated in X-VIVO™ 15 media, supplemented with M-CSF and IL-3. Non-adherent PSC-MC can be harvested from the supernatant of cultures and plated in X-VIVO™ 15 media+M-CSF without serum, or in RPMI media +10% serum+M-CSF, to generate PSC-MDM. A pure population of genetically modified PSC-MDM can be obtained by transducing PSC with a self-inactivating lentiviral construct (in this case expressing RFP-IRES-Puromycin^R^ under control of the EF1alpha promoter). Scale bar, 200 µM. Detailed protocol described in materials and methods. B) Characteristics of the defined and quick protocol. C) Histogram represents RFP expression by PSC-MDM differentiated from PSC transduced with EF1a-RFP-IRES-Puromycin^R^ (black line) or control PSC-MDM (shaded gray).

Two differentiation protocols were developed (Figure 1A+B). In the quick protocol, MDM were derived from human pluripotent stem cells (hPSC) cultured on a layer of mouse feeder cells, lifted mechanically to form variable-sized EBs by culturing the PSC for four days on ultra-low adherence plates. EBs were further differentiated by seeding approximately 20 EBs into a well of a 6-well plate cultured in X-VIVO™ 15 media supplemented with IL-3 and M-CSF.

In addition, a defined differentiation protocol was developed that uses solely feeder- and serum-free, fully defined products. This methodology would be required as a step to obtaining ‘good manufacturing practice’-grade PSC-myeloid cells for e.g. cell therapy, but is not essential for e.g. *in vitro* research purposes. PSC were grown on Synthemax™ plates, which are coated with synthetic extracellular matrix-derived cell adhesion-promoting peptides [9], [10], [11]. In preliminary experiments, we found that large EBs (10,000 cells) gave better yields of monocytes than smaller EBs. To generate uniform EBs from feeder-free PSC, a pre-defined number of enzymatically dissociated PSC were seeded into a round-bottomed Low-Attachment 96-well plate or an AggreWell™ plate, and uniform PSC aggregation was achieved by centrifugal force. The use of a 96-well plate allowed the isolation of a single EB per well (i.e. a useful format for medium-scale screening assays), whereas using AggreWell™ plates allowed larger scale production of EBs of defined size, generating 300 EB per AggreWell™. In order to induce haematopoiesis from these spin-EBs, the addition of three growth factors during the four-day EB formation was required: bone morphogenetic protein 4 (BMP-4), vascular endothelial growth factor (VEGF) and stem cell factor (SCF). EBs were then subjected to directed differentiation as described for the quick protocol.

Using either the quick protocol or the defined protocol, EBs adhered and within 2 weeks and produced a homogeneous population of PSC-MC that could be harvested weekly from the supernatant of the differentiation cultures. Harvested PSC-MC were cultured in M-CSF for 5–7 days to allow the differentiation into PSC-MDM. This final stage could be in either media (RPMI) supplemented with serum or using serum-free conditions (X-VIVO™ 15 media).

To explore the usefulness of the differentiation protocol to study MDM biology and pathogen interactions using a genetic approach, transgene expression in PSC-MDM was tested. PSC were transduced with a second generation, SIN lentiviral vector (LV-EF1α-RFP-IRES-Puromycin^R^). Cells were kept under continuous puromycin selection (5 µg/mL: a concentration at which untransduced cells died), thereby selecting a pure population of PSC containing the lentiviral vector. Fluorescent microscopy analysis showed RFP expression in PSC, which was maintained throughout the differentiation in the continuous presence of puromycin (Figure 1A, right panel). Flow cytometric analysis of RFP expression confirmed that a pure population of RFP-expressing PSC-MDM was obtained (Figure 1C), showing that continuous-selection during differentiation supports the generation of homogeneous-transgene-expressing PSC-MDM in this serum-free culture system.

**Figure 2:**
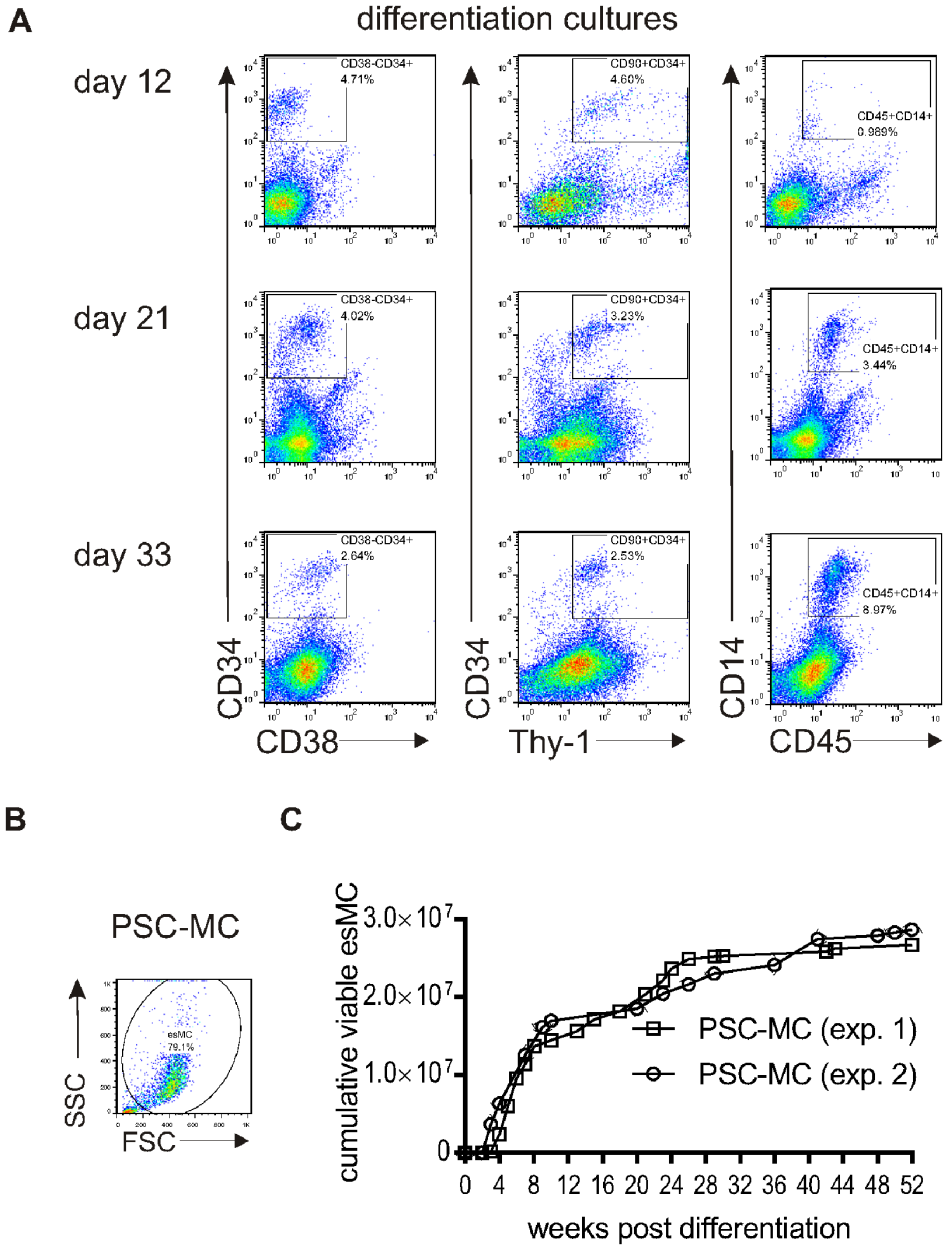
Monocytopoiesis characterization in differentiation cultures. A) Time-course analysis of cell surfaces marker expressed during monocytopoiesis. Adherent EBs were harvested at 12, 21 and 33 days after EB formation and stained for various cell surface markers. Immuno-fluorescence dot plot analysis of CD38 and CD34; Thy-1 and CD34; CD45 and CD14. Gates were determined by using the relevant isotype control antibodies. B) Forward Scatter (FSC) and Side Scatter (SSC) dot plot of harvested PSC-MC showing a gate around the homogenous cell population. C) Non-adherent PSC-MC were harvested from the supernatant of differentiation cultures and counted using a cell counter (Chemometec). The media were replaced for repeated PSC-MC harvests over a period of 52 week. Data represent the cumulative number of viable PSC-MC from 6 wells.

To begin to identify Haematopoietic Progenitors in the differentiation cultures, cultures were harvested at 12, 21 and 33 days after spontaneous EB formation and co-stained with CD34 and CD38, or CD34 and CD90 (Figure 2A, left and middle panel). The proportion of CD34^+^CD38^−^ and CD34^+^CD90^+^ cell populations (4.71% and 4.60%, respectively) were highest 12 days after EB formation and decreased subsequently, but were still present at 33 days. To identify myeloid lineage cells, differentiation cultures were co-stained with CD45 and CD14 (Figure 2A, right panel). CD45^+^CD14^+^ cells were low at 12 days after EB formation (0.99%) but increased over time (8.97%, 33 days after EB formation).

The differentiation cultures produced a homogenous population of PSC-MC, as illustrated by the cell scatter properties (Figure 2B), the gated ‘viable’ population being an average 85% ±2.7 (mean ± SEM, n = 16) of the total events. AO-DAPI stain confirmed that the PSC-MC had a mean viability ± SEM of 85% ±4.2, n = 7.

To monitor the long-term production of PSC-MC, PSC-MC were harvested routinely and yields were quantified (Figure 2C). Cumulatively, after 8 weeks of monocytopoiesis ≥1×10^7^ PSC-MC had been collected from a 6-well plate (57.7 cm^2^), and PSC-MC production could be continued for up to 1 year. Although yields decreased over time, 5×10^5^ CD14+CD45+ PSC-MC were still being produced per plate after 1 year of differentiation (Figure 2C and Figure S1). Similar results were obtained using induced-PSC lines (note the cumulative yield of monocytes was ∼10^7^ per plate over 3 months, approximately 2/3 that of HUES2, perhaps due epigenetic memory in the fibroblast-derived iPSC lines; Figure S2), and also using the fully-defined protocol (Figure S3).

### Analysis of Morphology and Phenotype of PSC-MC and PSC-MDM

**Figure 3:**
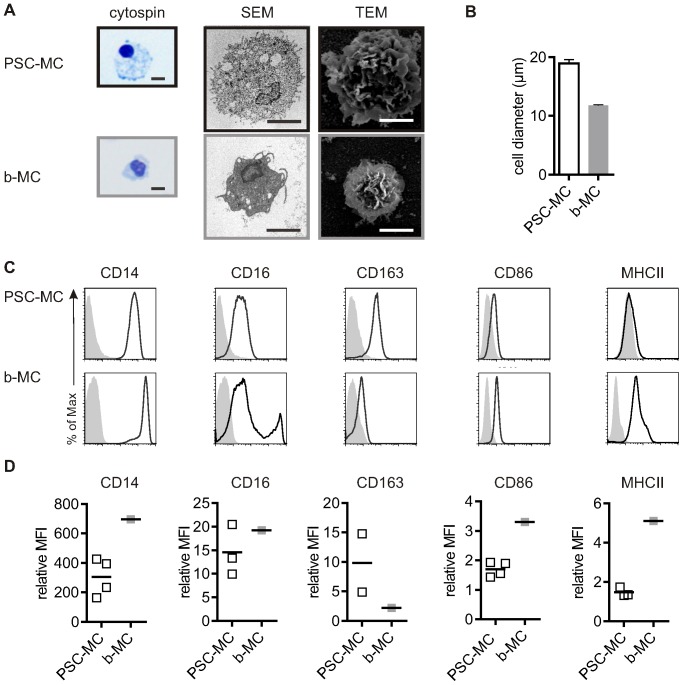
PSC-MC characterization compared to b-MC. A) Morphology of MC. From left to right: Eosin-methylene blue staining of MC from cytospins, Scanning Electron Microscopy, and Transmission Electron Microscopy. Scale bar, 10 µM. D) Diameter of PSC-MC (n = 7) and b-MC (n = 3) were determined by an AO-DAPI stain (Chemometec). Bars represent the mean diameter ± SEM. C) Phenotype of MC. Surface expression of CD14, CD16, CD163, CD86 and MHC II were measured by flow cytometry. Histograms represent surface staining (black line) compared to the isotype control (shaded gray). D) The symbols reflect the relative ratio of the geometric mean fluorescence intensity (MFI) over the isotype control of independent experiments.

PSC-MC morphology was generally similar to that of blood derived MC (b-MC), in that they were characterized by a single indented nucleus and a high cytoplasm-to-nucleus ratio (Figure 3A). All MC contained vesicles, but these were particularly large in PSC-MC, accounting for their relatively large diameter (mean ± SEM, PSC-MC: 18.9 µM ±0.70, n = 7; bMC: 11.8 µM ±0.14, n = 3) (Figure 3B).

To further validate the differentiation protocol and characterize the PSC-MC phenotype, the expression profile of a subset of myeloid cell surface receptors was assessed and compared to b-MC (Figure 3C+D). The LPS receptor CD14 was expressed at high levels on PSC-MC. The low affinity IgG receptor CD16 was expressed at low levels on PSC-MC, corresponding to the major b-MC population, which are thought to be less mature and less pro-inflammatory compared to the minor b-MC population, and which have lower antigen-presenting capacity [12]. The scavenger receptor CD163, which plays a homeostatic role in clearing haemoglobin and can also regulate cytokine production, was expressed at high levels in PSC-MC. The co-stimulatory molecule CD86 and antigen-presenting molecule MHC II were expressed at low levels on PSC-MC compared to b-MC.

**Figure 4:**
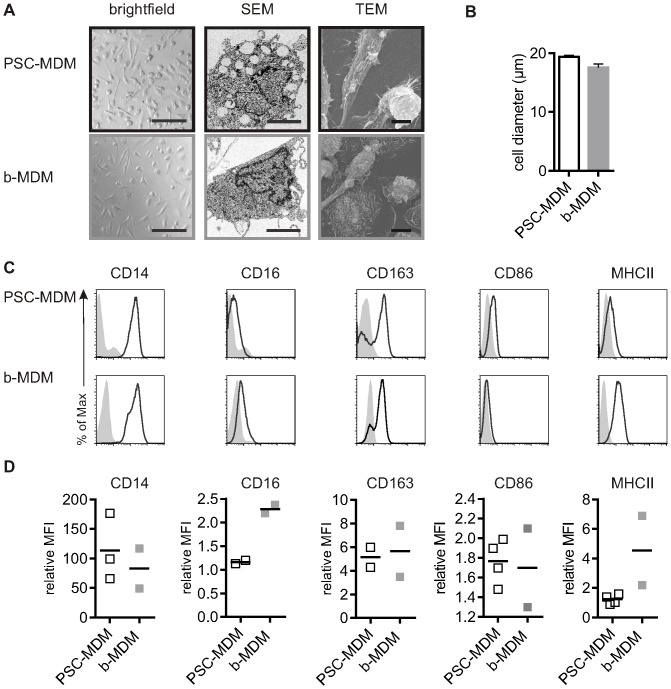
PSC-MDM characterization compared to b-MC. A) Morphology of MDM. From left to right: Representative brightfield image (scale bare 200 µM), Scanning Electron Microscopy and Transmission Electron Microscopy (scale bar, 10 µM) D) Diameter of PSC-MDM (n = 3) and b-MDM (n = 6) were determined by an AO-DAPI stain (Chemometec). Bars represent the mean diameter ± SEM. C) Phenotype of MC. Surface expression of CD14, CD16, CD163, CD86 and MHC II were measured by flow cytometry. Histograms represent surface staining (black line) compared to the isotype control (shaded gray). D) The symbols reflect the relative ratio of the geometric mean fluorescence intensity (MFI) over the isotype control of independent experiments.

To test whether PSC-MC can differentiate into MDM similar to b-MC, MCs were harvested and differentiated for 7 days in the presence of M-CSF, and their morphology and phenotype were assessed (Figure 4). PSC-MC differentiated into an adherent PSC-MDM population with characteristic membranes blebs and ruffles, with an b-MDM-like morphology. All MDM had a proportion of elongated (spindle-shaped), ‘M-MDM’-like cells (Figure 4A). M-MDM have been described as having greater anti-inflammatory, regulatory and phagocytic functions, versus ‘GM-MDM’, which have greater pro-inflammatory effects [13], [14], [15], [16], [17]. The diameter of PSC-MDM (mean diameter ± SEM: 19.4 µM ±0.06, n = 3) was similar to b-MDM (mean ± SEM: 17.6±0.60. n = 6), indicating that the size difference at the monocyte level was not apparent after a week of differentiation to macrophages (Figure 4B). Both PSC-MDM (mean viability ± SEM: 87.1% ±1.42, n = 3) and b-MDM were similarly viable (mean viability ± SEM: 90.6% ±1. 2, n = 6), as assessed by AO-DAPI staining and expressed expected macrophage markers, as assessed by FACs, although MHC Class II was notably low on PSC-MDM (Figure 4C,D).

Taken together, these results show that PSC-MC and PSC-MDM share morphological and phenotypical characteristics with b-MDM. Similar results were obtained using MC and MDM obtained from three induced-PSC lines from a normal donor (Figure S2), and using the fully defined protocol (Figure S3).

### MDM Derived from PSC are Functionally Similar to b-MDM

**Figure 5:**
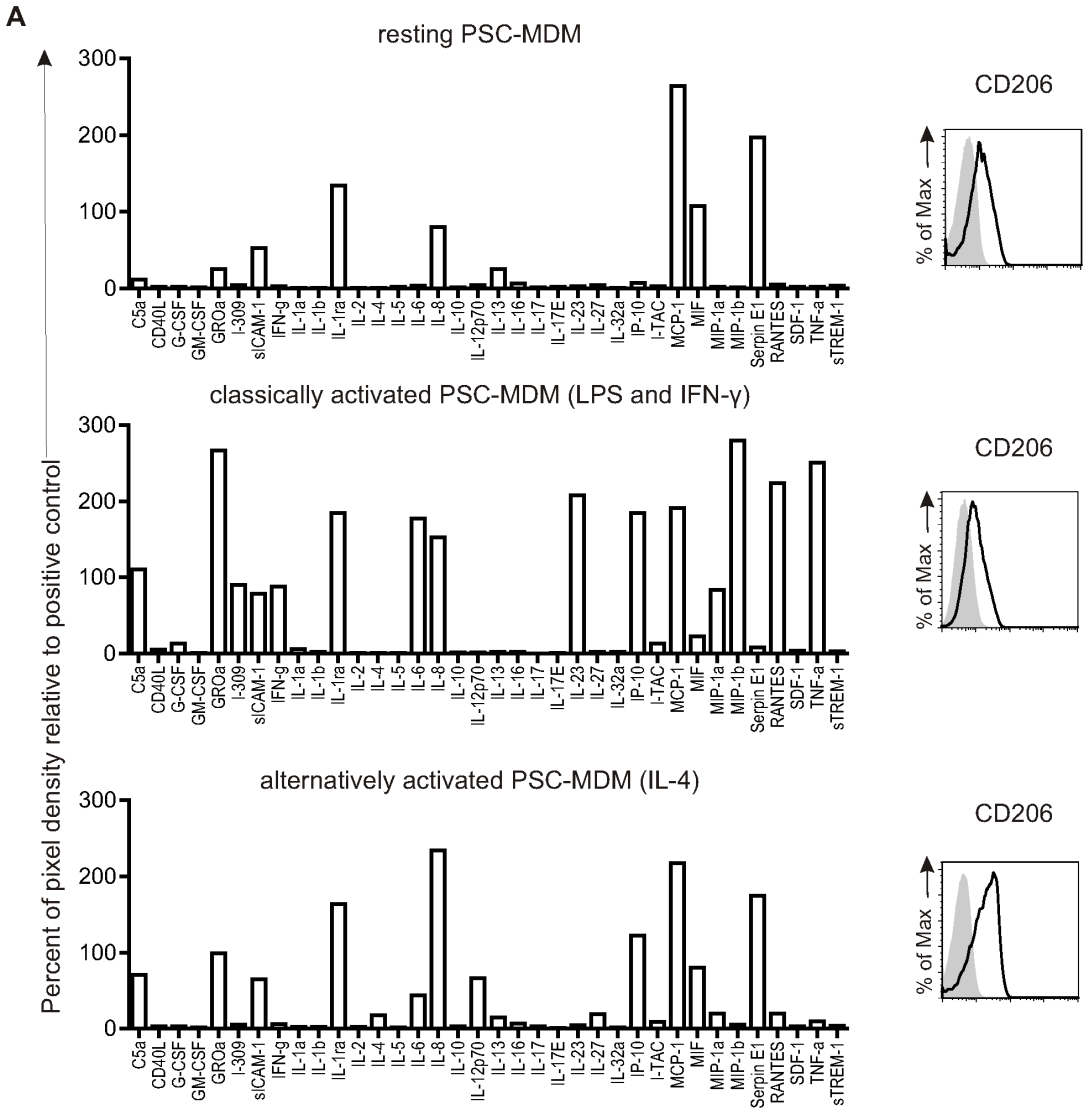
PSC-MDM activation. A) Cytokine production and surface expression of CD206 from resting (top), classically activated (middle) and alternatively activated (bottom) PSC-MDM. Histograms represent surface staining (black line) compared to the isotype control (shaded gray).

To assess whether PSC-MDM resemble b-MDM functionally, PCS-MDM were assayed for their ability to secrete cytokines and chemokines upon activation. Resting PSC-MDM (Figure 5, top panel) had a remarkably similar profile of cytokine production as b-MDM (b-MDM data shown in *Jiang et al.* [18], using exactly the same assay). As expected, cytokine secretion was upregulated upon classical activation (Figure 5, middle panel), but distinct from expression profiles of alternatively activated PSC-MDM (Figure 5, bottom panel) and from expression profiles reported for other cell types, such as T-cells [19], [20], [21]. As expected, IFN-γ, which was used to activate the cells, was detected at high levels in the supernatant by classically activated PSC-MDM, but only at background levels in resting or alternatively activated PSC-MDM. In agreement with their cytokine expression profile and mode of activation, classically activated PSC-MDM did not upregulate the mannose receptor CD206, unlike alternatively activated PSC-MDM [22]. The key pro-inflammatory cytokines TNF-α and IL-6 were secreted by the classically activated PSC-MDM. Also, key chemokines, such as IL-8, IP-10, MIP-1α, MIP-1β and RANTES (involved in attracting neutrophils, immature dendritic cells, natural killer cells, and activated T cells) were upregulated upon classic activation [23]. Classically activated PSC-MDM upregulated mostly the same cytokines as b-MDM albeit at variable levels [24]. Exceptions included the anti-inflammatory cytokine IL-10, which was secreted by b-MDM, but not by PSC-MDM. Murine alveolar macrophages have been shown to be unable to produce IL-10 in response to LPS, in contrast to peritoneal macrophages, so this finding is not without precedent, and may give some insight into the developmental status of these cells [25].

Overall, the results provide evidence of the functional similarity of PSC-MDM to b-MDM, with regard to cytokine production and response to classic activating ligands.

**Figure 6:**
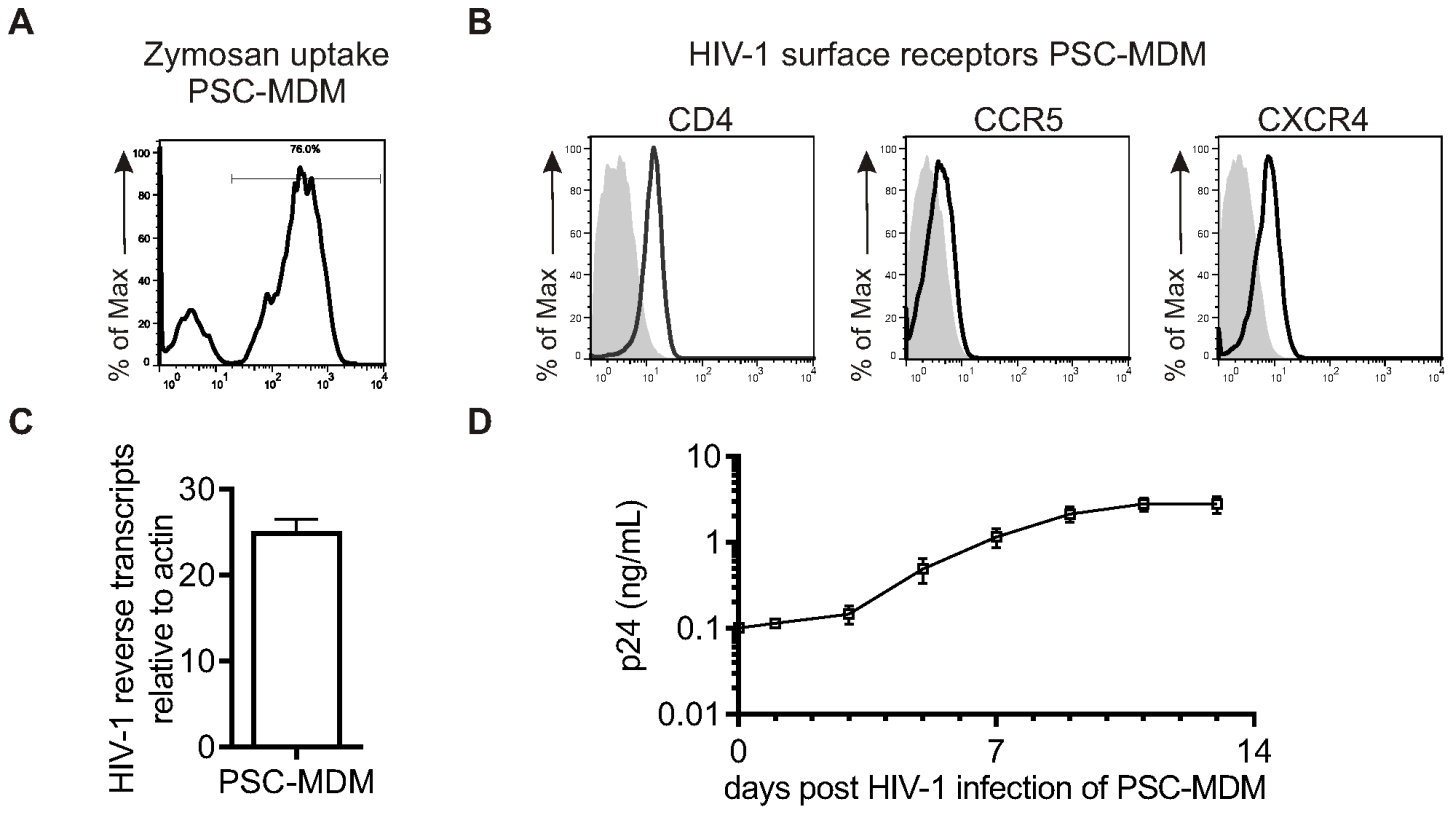
PSC-MDM pathogen interactions. A) Phagocytosis of ﬂuorescent zymosan particles. Representative histrogram showing percentage of zymosan-postive cells. B) Surface expression of the HIV-1 (co-) receptors CD4, CCR5 and CXCR4 were measured by flow cytometry. Histograms represent surface staining (black line) compared to the isotype control (shaded gray). C) Reverse transcription of HIV-1 was measured by detecting late (pol) products by q-PCR after 30 h of infection. Symbols represent the relative mean number of copies of HIV-1 DNA ± SEM of independent experiments (n = 3), normalised to the number of cells using a β-actin control. D) HIV-1 replication was measured by p24 ELISA. Symbols represent the mean p24 concentration at different time points ± SEM of independent experiments (n = 7).

Fluorescent yeast (zymosan) particles were used to assess the capacity of PSC-MDM to phagocytose. PSC-MDM were incubated with zymosan particles for 30 min at 37°C, surface-bound zymosan was quenched with trypan blue, and the percentage of cells that had taken up zymosan was quantified by flow cytometry (Figure 6A). PSC-MDM were highly phagocytic, with 77.8% ±1.2 of cells phagocytosing at least one particle in this time-frame (mean ± SEM,n = 3). These results indicate that PSC-MDM are highly competent at phagocytosis.

In order to assess whether PSC-MDM can be infected by HIV-1 and used as an *in vitro* model to study HIV-1 infection of MDM, HIV-1 infection and replication kinetics were investigated. HIV-1 infection requires CD4 expression, which functions as its main receptor. Depending on virus tropism, CCR5 or CXCR4 is required as the HIV-1 co-receptor. MDM are preferentially infected by R5-tropic viruses [26], [27], [28]. Firstly, CD4, CCR5 and CXCR4 expression on PSC-MDM was confirmed by flow cytometry (Figure 6B). Next, HIV-1 entry was assessed using the single cycle qPCR assay measuring HIV-1 late reverse transcripts (Figure 6C) and HIV-1 replication was measured by quantification of p24 antigen by ELISA (Figure 6D). Both HIV-1 assays demonstrated that PSC-MDM are permissive to HIV-1 infection and allow HIV-1 replication.

### Phenotype of iPSC Lines used in this Study

**Figure 7:**
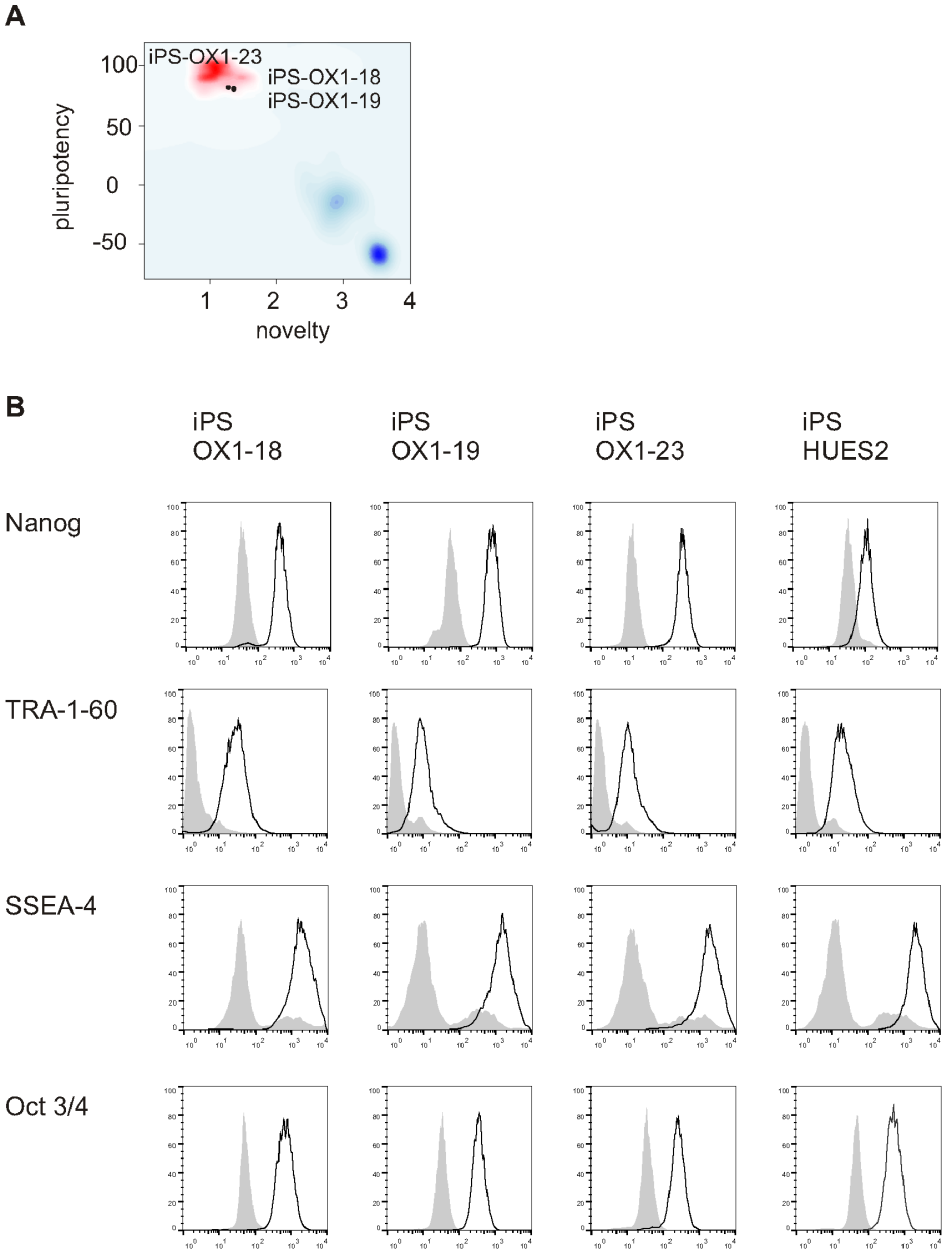
Phenotype of human Pluripotent Stem Cell lines. A) PluriTest analysis of Illumina HT12v4 transcriptome array data shows iPS-OX1-18, iPS-OX1-19 and iPS-OX1-23 to cluster with pluripotent stem cells (red cloud) and not with partly- or differentiated cells (blue clouds). Each circle represents one hiPSC line. B) Expression of human pluripotent stem cell markers. Surface expression of TRA-1-60 and SSEA-4, as well as total expression of Nanog and Oct 3/4, were measured by flow cytometry. Histograms represent surface staining (black line) compared to the isotype control (shaded gray).

Several of the standard characterisation assays for hiPSC lines are qualitative rather than quantitative. The iPSC lines in this study have been previously minimally assessed using these qualitative assays [24]. Here we present more detailed analysis of the lines. A more quantitative assessment of pluripotency has been recently developed, named Pluritest [29]. This is based upon analysis of transcriptome data from hiPSC and comparing gene expression profiles with a large reference set of genome-wide profiles from multiple cell and tissue types. We therefore analysed iPS-OX1-18, iPS-OX1-19 and iPS-OX1-23 using this more quantitative assessment. All three lines ‘passed’ this test and were therefore deemed to be fully reprogrammed and pluripotent (Figure 7A). Specifically, this test measures a) pluripotency score (expression of a set of genes associated with pluripotency, ergo high scores correlate with pluripotency). Relative scores were 31.38 (iPS-OX1-18), 30.06 (iPS-OX1-19) and 31.71 (iPS-OX1-23); b) ‘novelty’ score (expression of genes that would not be expected to be expressed in pluripotent stem cells, therefore this score inversely correlates with pluripotency (1.36, 1.35 and 1.28 were obtained, respectively). The Illumina HT12v4 transcriptome array results have been deposited in Gene Expression Omnibus (GEO) under accession number: GSE45470.

**Figure 8:**
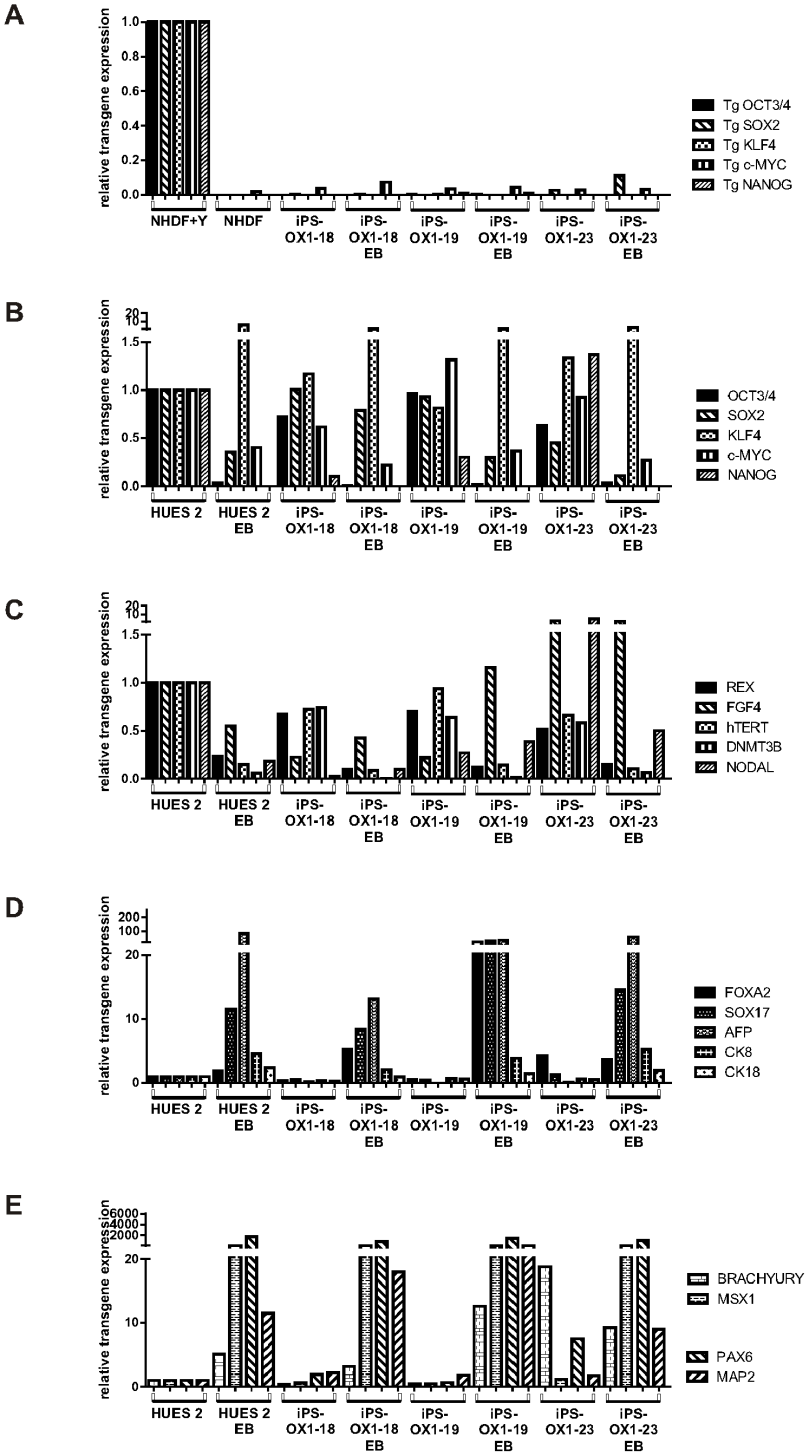
Further phenotype of human Pluripotent Stem Cell lines. Pluripotent stem cells were harvested after adaptation to matrigel/mTeSR™ and after undirected differentiation for 16d as embryoid bodies (10,000 cells per EB) and expression of transcripts was assessed by qPCR. A) Silencing of transgenes in iPS cell lines (normalised to NHDF transduced with Yamanaka reprogramming retroviruses 5d before harvest, ‘NHDF+Y’, relative to actin). B) Expression of endogenous version of reprogramming genes. C) Expression of additional endogenous pluripotency-associated genes. D) Expression of Endoderm-associated genes. E) Expression of mesoderm-associated genes (BRACHYURY and MSX1) and Ectoderm-associated genes (PAX6 and MAP2). B–E normalised to corresponding HUES2 transcripts (relative to actin).

The PSC lines were all shown by FACs analysis to express the pluripotency markers Nanog, TRA-1-60, SSEA-4 and Oct3/4 (Figure 7B), Quantitative RT-PCR showed that transgene expression was mostly silenced relative to transduced fibroblasts (Figure 8A), endogenous versions of reprogramming genes (Figure 8B) and additional pluripotency markers (Figure 8C) were expressed, and undirected differentiation to EBs showed downregulation of most of these markers (exceptions being Klf4 and FGF4), along with upregulation of a panel of markers representative of endoderm (Figure 8D), mesoderm and ectoderm (Figure 8E). Overall, the iPSC lines were demonstrably pluripotent.

**Figure 9:**
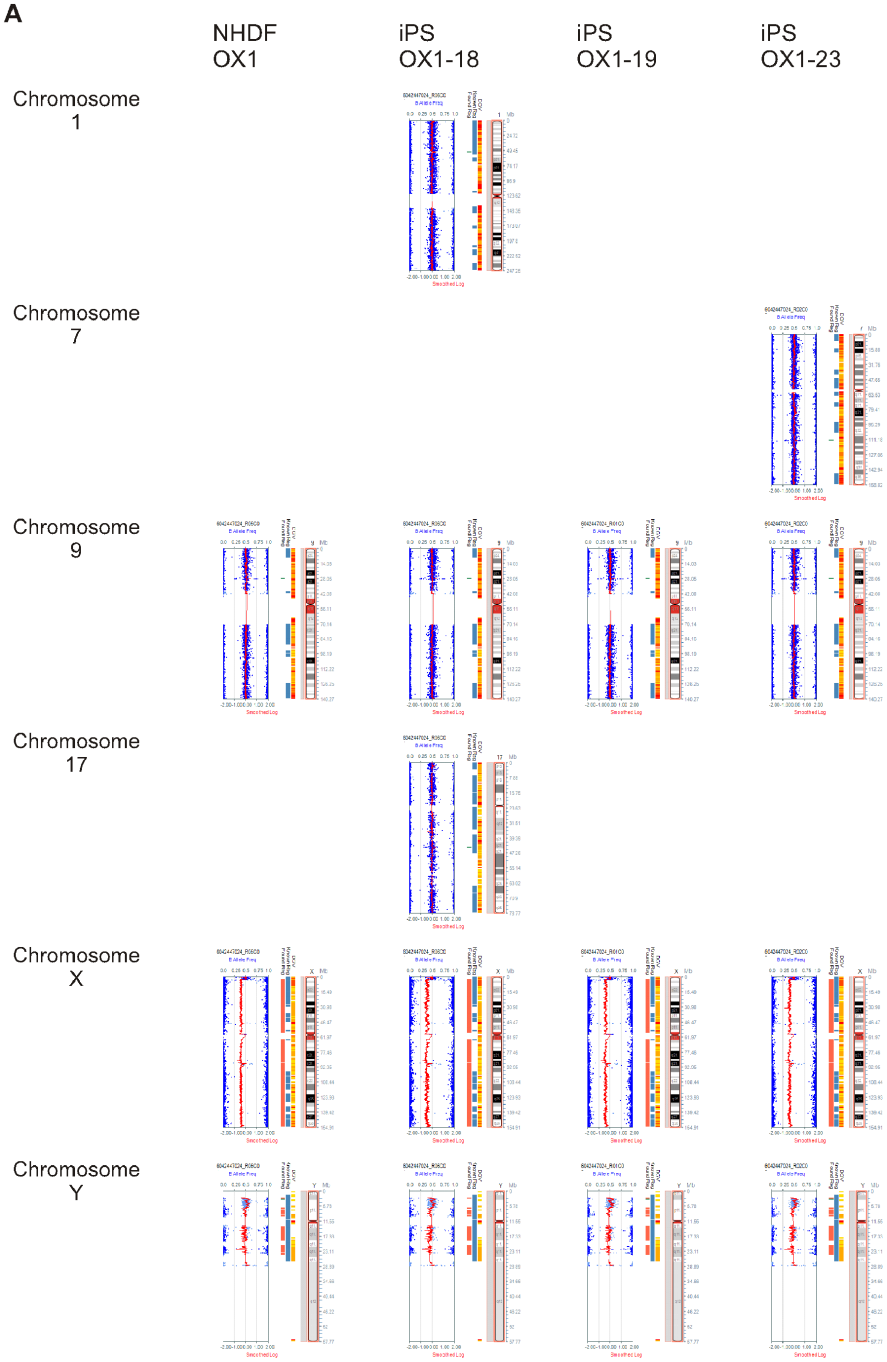
CytoSNP interrogation of human Pluripotent Stem Cell lines. KaryoStudio Detected Regions for NHDF-OX1 (parental fibroblasts) and derived hiPSC lines iPS-OX1-18, iPS-OX1-19 and iPS-OX1-23. The chromosomes which contained regions detected by KaryoStudio as deviating from expected (indicated in ‘Found Region’ column by green for amplification, orange for deletion) are shown, as are the X and Y chromosomes (which, being single copy, are Called despite being the expected copy number). Where an amplification has been detected, the SNP B allele frequency can be seen to show a corresponding small triploid region and the Smoothed Log R Ratio shows a small spike. Chromosomes that are not shown here were those (the majority) that had no regions detected by KaryoStudio as deviating from expected, and so are considered karyotypically normal. Note that the small changes detected here are well below the level of detection in other karyotype methods. The genes that these affect are listed in Table 1.

Analysis of the three lines by CytoSNP array revealed only extremely minor detected region differences between the parental fibroblasts and the derived lines. Low resolution KaryoStudio analysis was carried out previously [24]; here we supply higher resolution analysis of the minor SNP regions detected (Figure 9 and Table 1). The karyotype of the differentiated PSC-MC was shown by the same CytoSNP analysis to be almost indistinguishable from the PSC (Figure S4). GenomeStudio Cluster analysis of multiple hiPSC lines and parental fibroblasts generated at the JMSCF showed clustering of the OX1 hiPSC lines with the OX1 fibroblasts, indicating that they have indeed derived from these fibroblasts (data not shown). SNP datasets have been deposited in GEO under the accession number GSE45471.

**Table 1:** KaryoStudio Detected Regions Report for autosomes for NHDF-OX1 (parental fibroblasts), PSC lines and PSC-derived monocytes.

Sample ID	Chr	Start	Stop	Length	Confidence	Comment	CNV Index	Cytobands	# Markers	Genes
**NHDF-OX1**	9	28136460	28367812	231352	347	amplification, 1 gene	7	p21.1	24	LINGO2;
**iPS-OX1-18**	1	51428210	52943372	1515162	136	amplification, 21 genes	4	p32.3	186	RNF11; TTC39A; EPS15; EPS15; OSBPL9; OSBPL9; OSBPL9; NRD1; MIR761; RAB3B; TXNDC12; KTI12; BTF3L4; ZFYVE9; CC2D1B; ORC1L; PRPF38A; ZCCHC11; GPX7; FAM159A; C1orf163;
	9	28136460	28367812	231352	336		6	p21.1	24	LINGO2;
	17	44191085	44350293	159208	52	low confidence	8	q21.32	22	TTLL6; CALCOCO2; ATP5G1; UBE2Z;
**iPS-OX1-19**	9	28136460	28367812	231352	310		5	p21.1	24	LINGO2;
iPS-OX1-19 monocytes	9	28136460	28367812	231352	336		6	p21.1	24	LINGO2;
	14	106350394	106863833	513439	52	low confidence, no genes	7	q32.33	16	
**iPS-OX1-23**	7	111838957	112420072	581115	203	amplification, 6 genes	3	q31.1	34	IFRD1; IFRD1; C7orf53; C7orf53; TMEM168; C7orf60;
	9	28136460	28367812	231352	324		4	p21.1	24	LINGO2;
	17	44191085	44369335	178250	52	low confidence	6	q21.32	23	TTLL6; CALCOCO2; ATP5G1; UBE2Z; SNF8;
**HUES 2 Esc**	12	31195272	31401378	206106	113	amplification, 2 genes	7	p11.21	22	FAM60A; FLJ13224;
HUES 2 monocytes						no detected regions				

## Discussion

We have developed here serum-free and fully defined methods to produce consistent, large numbers of human pluripotent stem cell-derived monocytes, which resemble the major blood monocyte. PSC-MC were CD14^+^,CD16^low^ CD163^+^, and differentiated into functionally relevant macrophages similar to M-CSF-differentiated b-MDM [13], [30], [31], [32]. PSC-MDM were highly phagocytic and had similar resting and activated cytokine secretion profiles to b-MDM.

MHC II expression was notably low on PSC-MDM, which may suggest that PSC-MDM resemble immature macrophages [33], [34], [35]. Recent research, based on murine models, suggests that the majority of tissue macrophages arise in the embryo, before the development of HSCs and independent of the supply of blood-derived monocytes [34], [35], [36]. The PSC-MDM described here go through a monocyte stage, which argues against them representing yolk sac macrophages, but they may still represent an embryonic stage of development. As such they may represent a good model for tissue-specific macrophages, and further work will address their ability to be directed to differentiate to tissue-specific macrophages, such as microglia and kuppfer cells, by coculture/codifferentiation with neural or hepatic cell types respectively. This would provide useful in vitro models for examining the role of tissue-specific macrophages in, for example, neurodegeneration and liver damage.

The ability of the differentiation cultures described here to keep producing monocytes for a year is remarkable and could be harnessed for up-scaling PSC-differentiation cultures. Further work is needed to analyse the transcript profiles of monocytes produced over this time frame, and to identify the long-term progenitors in the cultures that enable this.

All the hESC and hiPSC lines and puro-resistant genetically modified lines we have tested (28 lines to date) have been capable of generating good numbers of monocytes using this serum-free protocol. The yield of puro-selected PSC-MC might be expected to be lower, given that cells that undergo silencing of the cassette during differentiation would die, but in the current set of experiments this was not observed (Figure S5). It should be noted, however, that it is likely that the site of integration and number of integrants will affect the degree of silencing, and we have generated cell lines for other studies where yields have been noticeably lower when under puromycin selection. Overall, this serum-free methodology compares favourably with our previously published protocol [37], where several hESC and hiPSC lines tested subsequently have produced poor yields. It is vital that differentiation systems be reproducible across multiple lines, with predictably high yields, to be able to use hiPSC-derived cells for studying phenotype and for drug screening, The system we have developed here fulfills these criteria and paves the way towards large-scale production of PSC-MDM for drug-screening studies. The quick method of EB production is simple and adequate where the aim is to generate large numbers of monocytes from multiple lines simultaneously for downstream assays. However, the EBs produced this way are variable in size, hence the alternate use of more controlled methods for EB generation (spin EBs) in the fully defined protocol. The fully defined protocol will be useful for investigating myeloid differentiation in more depth. It also paves the ways towards cGMP production of PSC-MC, as it allows for completely Xeno-free culture conditions, if mTeSR-2™ is used for the derivation and culture of the hiPSC.

We have demonstrated here the productive infection of PSC-MDM with strains of HIV-1 that use CCR5 as their co-receptor. This means they are useful as an in vitro model of HIV-1 infection of macrophages, especially as we also show that they can be genetically manipulated and then successfully differentiated using this serum-free differentiation protocol Note that the cultures require continuous selection with puromycin, which is necessary to ensure that the harvested monocytes have consistent expression of the transgene – without this, we have found that transgene expression drops to be detectable in only ∼10% of harvested cells, due to transgene silencing events. Note also that the expression level in the PSC-MDM is often lower than in the PSC, likely due to relative lower activity of the EF1α promotor in terminally differentiated MDM versus highly proliferative PSC – nonetheless, we are able to achieve physiologically relevant levels of expression of many transgenes. We are currently using this system in our laboratory to explore the entry pathway of HIV-1 into macrophages, as it enables knockdown of endogenous genes such as HIV-1 receptors, and reconstitution with engineered versions containing targeted amino acid substitutions – manipulations that are not practicable using b-MDM. PSC-MDM offers a robust approach, illustrated by the fact that other groups have used PSC-MDM, derived using their own protocols, for HIV-1 studies [4], [5]. The lentiviral method we describe here for introducing transgenes is appropriate for delivery of small genes under the control of small promotors, that can be packaged within the packaging capacity of lentiviruses. For more physiological control of expression, specific editing of endogenous genes within the genome of PSC could be undertaken – however, this is technically still quite challenging and slow, requiring rounds of positive and negative selection and screening of multiple clones [38].

In summary, the methods we describe here for efficient, scalable production of genetically-modifiable macrophages from human pluripotent stem cells, using defined media, represent a powerful addition to the repertoire of methods available to investigate macrophage biology.

## Materials and Methods

### Ethics Statements for use of Adult Blood and Stem Cell Lines

Adult human blood was obtained from anonymous donors through the UK National Blood Service and tested negative for HIV-1, hepatitis B/C, and syphilis. Local Institutional Review Board approval was sought for this work from Oxford University’s Central University Research Ethics Committee (CUREC), and we were informed that specific ethical approval was unnecessary for this study, in accordance with their guidelines on the use of human blood. However there are occasions when the National Blood Service donating the buffy coats may require ethical approval from the University. In this instance a checklist completion will suffice. Applicants should answer Question C (8) as a ‘NO’. A covering note should be sent to the Secretary of the MSD IDREC with the checklist explaining that the research uses buffy coats and the NBS requires University ethical approval.” Although not required by NBS, we completed a checklist as indicated and received exemption from MSD IREC.

The human ES cell line HUES-2 (passages 16–38) was obtained from the HUES Facility, University of Harvard [39]. Ethical approval for work on all hES cell lines was reviewed and approved by the UK Stem Cell Bank Steering Committee (Medical Research Council, London UK, 20.10.2005, who specifically approved this part of the study).

The human hiPSC lines iPS-OX1-18, iPS-OX1-19 and iPS-OX1-23 (See Methods S1), were all derived in-house from fibroblasts from a skin biopsy after signed informed consent (Ethics committee: National Health Service, Health Research Authority, NRES Committee South Central – Berkshire, UK, who specifically approved this part of the study - REC 10/H0505/71).

### Cell Count, Viability and Diameter

Cells were counted and assessed for diameter and viability using NC-3000 Viability and Cell Count Assays (chemometec). 1 µL of solution 13, containing AO and DAPI (chemometec) and 19 µL of cell suspension were mixed and from this 9 µL was loaded onto a chamber of a A8-slide (chemometec). All viable cells are stained with AO and non-viable cells are stained with DAPI. Alternatively, cells were counted and assessed for viability manually using trypan blue dye and a haemocytometer.

### PSC Culture

PSC were cultured at 37°C with 5% CO2. To achieve feeder-free, serum-free PSC culture, PSC were cultured on Synthemax™ plates^.^ (Corning). PSC were maintained in 2 mL mTeSR™-1 medium (Stem Cell Technologies) and passaged when colonies covered approximately 80% of the culture surface. To passage cells, the media was removed, PBS was added to wash cells, and 1 mL Collagenase IV (1 mg/mL, 200 U, Life Technologies, Invitrogen) was added and incubated for 4 min at 37°C. Next, the collagenase IV was removed, cells were washed with PBS and 2 mL of warm mTeSR™-1 medium was added. To lift the cells, the cells were gently scraped and pipetted up and down to break up clumps to pieces 0.5–1 mm (not single cells) which were plated at ∼10,000 cells/cm^2^ (corresponding to ∼1∶4 split). The passaged PSC was returned to incubator and not fed for the first 48 hours, but thereafter fed daily with fed 2 mL media (full media change). Alternatively, PSC were cultured on Mitomycin C-inactivated mouse embryo fibroblasts (MEFs) in PSC media. PSC media consisted of knock-out DMEM (Invitrogen), 10% knock-out-Serum Replacement (Invitrogen), 2 mM Glutamax-I (Gibco), 100 U/mL penicillin (Invitrogen), 100 µg/mL streptomycin (Invitrogen), 1% non-essential amino acids (Invitrogen), 0.055 mM β-mercaptoethanol (R&D), 10 ng/mL bFGF (R&D), 5 µg/mL heparin (Sigma) and 0.5% Albumin (Sigma). MEF’s were prepared the day before on 0.1% gelatin-coated tissue culture plates (Corning) at a density of 50,000 cells/cm^2^ in PSC media. The following day, PSC were plated at a density of 5,000–10,000 cells/cm^2^ and supplemented with 1 mM Rock-inhibitor (Y27632; Calbiochem). Medium was changed daily by removing 50% of the media and replacing with fresh PSC medium. Colonies were passaged every 5 days by manual micro-dissection.

### Genetic Modification of PSC

For constitutive expression of a transgene, the coding sequence for the Red Fluorescent Protein was inserted into the self-inactivating 2^nd^ generation lentiviral vector backbone plasmid pEF1-IRES-Puro-WPRE-SIN (based on ([40], [41]). The inserted gene was, therefore, under the control of the constitutively active Elongation Factor 1α (EF1α) promotor, and was transcribed on a bicistronic transcript together with the Internal Ribosome Entry Sequence (IRES), followed by the gene for resistance to puromycin. At the ribosome, the two genes on the bicistronic transcript are translated as two separate peptides; however, the above arrangement ensures that cells expressing the first gene can be reliably selected with puromycin. Lentivirus was prepared as described previously [24]. Lentivirus was added to PSC (∼70% confluence) with a MOI of approximately 0.08 in a total volume of 1 mL, supplemented with polybrene (5 µg/mL, Sigma) onto one well of a 6-well plate and centrifuged for 1 h 37°C at 1600 *g*. PSC were incubated at 37°C for 3 h after which the inoculum was replaced with fresh media. PSC were fed as normal for 2 days, and on day 3 puromycin (5 µg/mL, Sigma) was added.

### EB Formation by Mechanical Dissociation

PSC were mechanically dissociated by scoring each 10 cm^2^ well into a grid of approximately 100 patches with a 23G needle. Next, the patches were lifted with a cell scraper. Patches were transferred into a well of a 6-well ultra-low adherence plate (Corning) in PSC culture medium and cultured for 4 days in PSC media. All results shown use EBs formed by this method, except where specifically stated.

### EB Formation by Spinning

Spin-EBs were formed using either a 96-well ultra-low adherence plate (Costar 7007) or AggreWells^TM^800 (Stemcell Technologies). In both cases, PSC were washed with PBS and harvested by incubating the cells for 5 min at 37°C with 1 mL of warm TrypLE Express (Gibco by Life Technologies). The cells and TrypLE were well mixed into a single cell suspension by pipetting up and down and collected in a 15 mL centrifuge tube and diluted 1∶10 with PBS. Cells were counted and spun down. After centrifugation PBS was aspirated and the cell pellet was tapped loose and resuspended in mTeSR™-1 spin-EB medium. mTeSR™-1 spin-EB medium consisted of mTeSR™-1 (Stem Cell Technologies), supplemented with 1 mM Rock-inhibitor (Y27632; Calbiochem); 50 ng/mL BMP-4 (Peprotech EC Ltd.), 20 ng/mL SCF (Miltenyi Biotec Ltd), 50 ng/mL VEGF (Peprotech EC Ltd.). For generating EBs in 96-well ultra-low adherence plates (Costar 7007), cells were resuspended at 10 µL/mL to give a final cell concentration of 1×10^5^ cells/mL and 100 µL was added per well. The 96-well ultra-low adherence plate was centrifuged at 800 rpm for 3 min and the plate was gently put into the incubator and left for 4 days. EBs were fed at day 2 by gently aspirating 50 µL medium and very gently adding 50 µL of fresh EB medium dripping down the side of the well so the EBs are not disturbed. To harvest EBs at day 4, the contents of the wells were pipetted up and down several times to dislodge the EBs from the micro-wells. The EBs were transferred to a tissue culture plate, which was then tilted to remove media without removing EBs (which settle to the edge). Differentiation media was added, as described below. For AggreWells^TM^800 (Stemcell Technologies, 300 micro-wells), plates were first prepared by rinsing each AggreWell with PBS and aspirating the PBS. Next, 1 mL of mTeSR™-1 spin-EB medium was added to the AggreWell (which not yet contained cells). The AggreWell plate with medium, but no cells, was centrifuged at 3000 g for 1–2 min (insuring centrifuge is balanced), to remove microscopic air bubbles. After preparing the plate, 1 mL of 4×10^6^ PSC were added per well. The plate containing PSC and 2 mL of spin-EB medium per well was centrifuged at 800 rpm for 3 minutes. The plate was examined under the microscope to verify cells were evenly distributed among the micro-wells. Very gently the plate was put into the incubator and left for four days. The EBs were fed daily with spin-EB medium (first brought to RT), by gently aspirating 1 mL medium using a p1000 Gilson and very gently adding 1 mL fresh spin-EB medium in a drop-wise manner down the side of the well so the EBs were not washed out of the microwells. This wash was repeated to achieve a 75% medium change overall. To harvest EBs at day 4, the contents of the wells were pipetted up and down several times using a 5 mL serological pipette to dislodge the EBs from the micro-wells. The contents was taken up and transferred onto a 40 µM cell strainer inverted over a 50 mL centrifuge tube. The contents were washed out of the well several times with PBS and transferred onto the same strainer to collect all EBs and wash the EBs on the strainer. This way, the EBs were on top of the inverted strainer and the media/PBS passed through the inverted strainer into the 50 mL collection tube. The inverted strainer, with the EBs balanced on top, was carefully inverted over onto a new 50 mL centrifuge tube so that the EBs were now at the bottom of the strainer and could be collected into the new 50 mL tube, by passing though differentiation media (contents of media described in section 2.1.6). The strainer was held at an angle to facilitate the collection of EBs. EBs could be counted by transferring 50 µL sample to a well of a 96-well plate and using a light microscope.

### Directed Differentiation of EBs to Produce esMC

For differentiation, ∼10 EBs were transferred into one well of a six-well tissue culture plate in 3 mL medium or 1 EB was transferred into one well of a 24-well tissue culture plate in 1 mL medium. 2/3 of the medium was replaced every 5 days. Culture medium consisted of X-VIVO^TM^15 (Lonza), supplemented with 100 ng/mL M-CSF (Invitrogen), 25 ng/mL IL-3 (R&D), 2 mM glutamax (Invitrogen), 100 U/mL penicillin and 100 µg/mL streptomycin (Invitrogen), and 0.055 mM β-mercaptoethanol (Invitrogen). Once esMC were visible in the supernatant of the cultures (from 2–3 weeks onwards), non-adherent esMC were harvested weekly from the supernatant of EB cultures. For characterization of surface antigen during monocytopoiesis, adherent EB cultures were harvested using Collagenase B (Roche). Collagenase B was obtained as a powder and dissolved in PBS at 0.4 U/mL and sterilised by passing through a 0.22 µM filer. The media of cell cultures was removed and cultures were washed with 2 mL PBS. 1 mL of Collagenase B was added per 9.5 cm^2^ (1 well of a 6-well plate) and incubated at 37°C for 1–1.5 hours. Next, with a pair of syringe needles (26 g × ½”) cell cultures were pulled apart and another 0.5 mL collagenase B was added. The cell suspension was pipetted up and down to further break up clumps and returned to the incubator for another 30 min. Next, the cell suspension, was passed through a 100 µM cell strainer, followed by PBS, and cells were counted and prepared according to the methods for flow cytometry.

### Blood-derived Monocytes Isolation and Monocyte Purification

Peripheral blood mononuclear cells (PBMCs) were isolated using Ficoll-Paque Plus (GE healthcare) density gradient centrifugation from donated blood. After centrifugation (2000 rpm for 20 min, RT, brake off), the layer of PBMCs were isolated and washed (1500 rpm for 7 min, 4°C, brake low) five times in PBS (PAA) and until essentially free of erythrocytes and granulocytes. PBMCs were used for CD14^+^ cell isolation using anti-human CD14 magnetic beads (Miltenyi Biotec, 130-050-201) according to the manufacturer’s instructions.

### Macrophage Culture and Maturation

Monocytes were plated onto tissue culture-treated 6-well plates (Corning) at a density of 1.5×10^6^ cells/well; or at 6×10^5^ cells/well of a 12-well plate (Greiner Bio-One); or at a density of 3×10^5^ cells/well of a 24-well plates (Corning), or 1×10^5^ cells/well of a 96-well plates (Corning). Monocytes were maintained in MDM medium (.375×10^6^/mL), consisting of RPMI 1640 (PAA) with 10% FCS (PAA), or in X-VIVO^TM^15 (Lonza) where specifically stated. The medium was supplemented with 100 ng/mL (approx. 1.7×10^4^ units/mL) recombinant human M-CSF (R&D Systems), as well as, 2 mM glutamine (PAA), 100 U/mL penicillin and 100 µg/mL streptomycin (PAA). Monocytes were incubated at 37°C, with 5% CO_2_ and differentiated for 5–7 days prior to use. For activation of macrophages for cytokine profile experiments, IFNγ (R&D, 100 U/mL) and LPS (Sigma, 100 ng/mL), or IL-4 (50 ng/mL, R&D) were added to the culture medium during the last 16 hours of culture. For MDM analysis, cells were detached using cold PBS containing EDTA (5 mM).

### Microscopy

Light images were taken using a Zeiss Axiovert 40 inverted microscope. To capture images, a digital camera (Colour CMOS UXGA [2.0 megapixel] USB 2.0 Camera; XL imaging) and QImaging software was used.

For Scanning Electron Microscopy (TEM), monocytes were plated onto 13 mm coverslips and differentiated into macrophages, as described above or monocytes were settled onto coverslips coated with 1% polyethyleneimine. Cells were fixed with 2.5% glutaraldehyde, 2% paraformaldehyde in 100 mM cacodylate buffer (pH 7.2) O/N at 4°C. Samples were post-fixed with 1% osmium tetroxide in 100 mM cacodylate buffer (pH 7.0) for 1 h at RT. Next, cells were washed with distilled water and dehydrated with 50% ethanol for 15 min.; 70% ethanol O/N; 90% ethanol for 15 min.; and finally 100% ethanol for 3 times 30 min. Next, samples were dried using liquid CO_2_ in an Autosamdri®-815 (Touismis) critical point dryer and mounted onto double-sided carbon tabs on aluminium SEM stubs and coated with gold in an E5100 Series II sputter coater (Bio-Rad). Cells were examined using a JOEL JSM 6390 scanning electron microscope.

For Transmission Electron Microscopy (TEM) monocytes were plated onto 13 mm coverslips and differentiated into macrophages, as described above or monocytes were used directly. Cells were fixed with 2.5% glutaraldehyde, 0.1% tannic acid in 100 mM cacodylate buffer (pH 7.2) for 16 h at 4°C. Cells were washed by incubating the samples with 200 mM cacodylate buffer (pH 7.2) for three times 10 min. Samples were post-fixed with 1% osmium tetroxide in 100 mM cacodylate buffer (pH 7.0) for 1 h at RT. Next, cells were washed with distilled water. Cells were washed with distilled water and dehydrated with 50% ethanol for 15 min.; 70% ethanol for 15 min.; 90% ethanol for 15 min.; and finally 100% ethanol for 3 times 30 min. Cells were pelleted by centrifugation. Samples were embedded using Epoxy resin (Agar 100, Agar Scientific). To allow the Epoxy resin to infiltrate the sample, samples were incubated for 1 h with ethanol and resin (added in a 2∶1 ratio); 1 h with ethanol and resin (added in a 2∶1 ratio); 1 h with ethanol and resin (added in a 1∶2 ratio); O/N with resin. The next morning, the resin was replaced with fresh resin and incubated for 8 h. Next, the samples were put into embedding moulds with fresh resin. Samples were polymerised for 24 h at 60°C and sectioned using a Reichert Ultracut E microtome and post-stained with 2% uracil acetate and Reynold’s lead citrate [42]. Samples were examined using a FEI Tecnai 12 electron microscope at 120 kV.

### Cytospins

For cytospins, 1.0×10^5^ cells were centrifuged (Shandon Cytospin) at 400 rpm for 5 min to make cytospins. For eosin-methylene blue staining, slides were fixed in methanol for 30 sec, stained with methylene blue for 30 sec and stained with eosin for 1 min, and washed with water. Slides were mounted with round cover slips using DPX and dried overnight. Images were taken using a Nikon Coolscope.

### Flow Cytometry

For analysis of cell surface molecules, 0.5–1×10^6^ cells were washed and stained in flow cytometry buffer consisting of PBS, human IgG (10 µg/mL Sigma), FCS (1% Hyclone) and sodium azide (0.01%), with an antibody or an isotype-matched control (with same fluorophore, from the same manufacturer) on ice for 30 min. For two-colour staining, two antibodies or two isotype controls (attached to different fluorophores) were added together. For total staining, cells were first fixed with 4% paraformaldehyde in PBS and permeabilised using 0.1% saponin (Sigma) in flow cytometry buffer. Antibodies used are described below. After the primary staining, cells were washed three times and if unconjugated antibodies were used, stained with a secondary antibody on ice for 30 min and washed another three times. Cells were fixed with 4% paraformaldehyde in PBS. Fluorescence was measured using a FACS Calibur (Becton Dickinson), and data was analysed using FlowJo software on marked cell populations on FSC-SSC dot plots.

The following antibodies (clone, isotype controls, supplier) were used: CD34-APC (4H1, IgG1κ -APC, eBioscience), CD38-PE (IB6, IgG2b-PE, Miltenyi Biotec Ltd), Thy-1-PE (5E10, IgG1κ-PE, BD), CD14-APC (MEM-15, IgG1-APC, Immunotools), CD45 (MEM-28, IgG1-APC, Immunotools), CD16 (LNK1, IgG1-APC, Immunotools), CD163-PE (GHI/61, IgG1κ-PE, BD), CD86 (BU63, IgG1-FITC, Immunotools), MHCII (MEM-12, IgG1-FITC, Immunotools), CD206 (19.2, IgG1κ-PE, eBioscience), CD4-APC (11830, IgG2a-APC, R&D Systems), CCR5-PE (45531, IgG2b-PE, R&D), CXCR4 (44717, IgG2b-PE, R&D), TRA-1-60 (B119983, IgM-488, Biolegend), SSEA-4-633 (MAB1435, IgG3-488, R&D), Oct 3/4-488 (MAB1759, IgG2b-488, R&D), Nanog-488 (AF1997, IgG-488, R&D).

### Phagocytosis Assay of Zymosan Particles

To measure phagocytosis of *S.cerevisiae* Zymosan A BioParticles® (Alexa Fluor 488 conjugated, Invitrogen), the BioParticles were reconstituted according to the manufacturer’s instructions. A ratio of 2 particles per macrophage was added in serum-free RPMI. Phagocytosis was allowed to occur for 30 min at 37°C, followed by 1 wash with PBS, 1 wash with 250 µg/mL trypan blue in PBS to quench particles bound to the outside of the cell, and a further wash with PBS. Cells were then incubated with 0.25% trypsin/0.5 mM EDTA (in Hanks’ Balanced Salt Solution; Sigma) at 4°C for 1 h to detach the cells. Detached macrophages were centrifuged at 400 *g* and fixed with 4% formaldehyde in PBS. Uptake of zymosan was quantified using a Becton-Dickinson FACS Calibur flow cytometer and data analysed using FlowJo software. Fluorescence negative cells (not fed zymosan) were used to establish a threshold for quantifying the percentage of positive cells having taken up one or more zymosan particle.

### HIV-1 Assays

HIV-1 BaL was obtained from *Gartner et al.* from the AIDS Research and Reference Reagent Program, Division of AIDS, NIAID, NIH and amplified in PBMCs for 12–21 days before harvesting [43].

To measure HIV-1 late reverse transcripts by qPCR, HIV-1 BaL viral stocks were treated with 100 µg/mL DNase I (Sigma). Macrophages were spinoculated with 500 µL DNase I treated HIV-1 BaL by centrifugation at 2000* g* for 90 min at 37°C and incubation for an additional 30 min at 37°C. The inoculum was removed and replaced with fresh media. After incubating the cells for another 28 h in the incubator, media was removed and the cells (still on tissue culture plates) were stored at −20°C or used directly to extract DNA. DNA was extracted using DNeasy Blood and Tissue Kit (Qiagen) according to the manufacturer’s instructions, except PBS and proteinase K were directly added onto the cells on the plates and cells were harvested from plates by pipetting up and down. DNA was eluted in AE buffer, supplied with the kit and used in a Taqman real-time qPCR reaction using Brilliant QPCR Core Reagent Kit (Agilent Technologies) with the following primers and probe:

5′–3′: TGGGTTATGAACTCCATCCTGAT (forward primer),

5′–3′: TGTCATTGACAGTCCAGCTGTC (reverse primer), and

5′–3′: FAM-TTTCTGGCAGCACTATAGGCTGTACTGTCCATT-TAMRA (probe).

The primer set used detects a conserved HIV-1 cDNA sequence, corresponding to a 81-bp fragment from the HIV-1 reverse transcriptase gene *(pol)*. Reactions contained 10 µL BrilliantqPCR Mastermix (Agilent Technologies), 375 nM forward primer, 375 nM reverse primer, 125 nM probe, 300 nM reference dye and ∼80 ng DNA from infected cells (generally 2 µL), in a total reaction volume of 20 µL. Standards were prepared in duplicate using the pNL4.3.Luc.R-E- HIV-1 backbone plasmid diluted ranging from 10 to 1×10^7^ copies. To allow normalisation of the number of copies of HIV-1 DNA to the number of cells, β-actin DNA was quantified (β-actin control kit, Eurogentec) in parallel, according to the manufacturer’s instructions. β-actin standards were prepared using human Xsomal genomic DNA (Eurogentec) at 10 fold dilutions ranging from 60 ng to 0.006 ng. The fast qPCR programme was as follows: 95°C for 3 min, and 40 cycles of 95°C for 5 sec, 60°C for 10 sec, and performed on an Applied Biosystems StepOne Plus Real Time PCR machine, with StepOne software. To measure multiple rounds of HIV infection, macrophages were infected in 96-well plates with HIV-1 BaL for 6 h at 37°C. The inoculum was removed and replaced with RPMI +10% FCS +100 ng/mL M-CSF. Over a 2–3 week period, supernatant samples were taken at intervals and kept at −20°C. Samples were diluted in TES (1×Tris buffered saline TBS, 1% Empigen Fluka, 10% FCS, 0.05% Tween 20), heat inactivated and p24 levels quantified by p24 ELISA as described previously [44].

Supernatant samples were taken at intervals and kept at −20°C. 96-well flat-bottomed high binding plates (Greiner) were coated with 100 µL/well of 10 µg/mL anti-p24 (D7320, Aalto) in coating buffer (0.05 M carbonate-bicarbonate buffer pH 9.6, Sigma) O/N at RT, rocking. Plates were then washed 4 times in PBS with 0.05% Tween 20 and blocked with 200 µL 2% BSA (Sigma) in TBS (Tris buffered saline, 0.144 M NaCI, 0.05% Tween 20 (Sigma), 25 mM Tris-HCI, pH 7.5) for 30 min at RT, and the washing steps were repeated. A standard curve was created with dilutions of recombinant HIV-1 p24 protein (Aalto) in TES (TBS +1% empigen (Fluka), 10% FCS) to provide concentrations ranging from 200 ng/mL to 0.23 ng/mL. Samples from infected cells were diluted 1 in 4 in TES to give a final concentration of 1% empigen and heated to 56°C for 30 min to inactive the virus.

100 µL of the sample or standard was applied to the plates in triplicate. The plate was covered and incubated for 2 h at 37°C with gentle agitation (75 rpm). Plates were washed 5 times as before and 100 µL of biotinylated anti-p24 antibody (Aalto BC1071-BIOT) diluted in TT/SS (TBS, 20% FCS, 0.05% Tween 20) was added for 2 h. Plates were washed as before, and 100 µL of streptavidin-HRP (Pierce) diluted 0.1 µg/ml in TT/SS applied for 1 h. Plates were washed and then developed with 100 µL 1-step Ultra TMB-ELISA (Pierce, #34028) according to the manufacturer’s instructions. The reaction was stopped with 100 µL 1M H_2_SO_4_ (Sigma) and the absorbance was read at 450 nm using a Molecular Devices Emax precision microplate reader and SoftMax Pro software version 4.0. The data were analysed using GraphPad Prism software to create standard curves (2-site binding equation) and to calculate unknown values.

### Pluritest

After converting hiPSCs to feeder-free culture on Matrigel (BD Biosciences) for at least 3 passages, cells were dissociated with TryplE (Life Technologies) and harvested. RNA was extracted from iPS-OX1-18 (passage 12), iPS-OX1-19 (passage 10) and iPS-OX1-23 (passage 12), using an RNeasy kit (Qiagen), for Illumina HT12v4 transcriptome array analysis. The data files were then uploaded to www.pluritest.org and scored for pluripotency, as previously described [29].

### Quantitative RT-PCR for Assessment of Pluripotency and Differentiation Transcripts in PSC Lines

After converting hiPSCs to feeder-free culture on Matrigel (BD Biosciences) for at least 3 passages, cells were dissociated with TryplE (Life Technologies) and either harvested for quantitative RT-PCR analysis [HUES 2 (passage 32), iPS-OX1-18 (passage 12), iPS-OX1-19 (passage 10) and iPS-OX1-23 (passage 12)], or seeded in mTeSR-1 medium (Stem Cell Technologies) supplemented with Rock inhibitor (10 µM; Calbiochem,) into Aggrewell plates (Stem Cell Technologies) at 10,000 cells per EB. EBs were cultured for 8 days in suspension and then 8 days on gelatin, following the protocol of Takahashi et al. [45], then harvested for qRT-PCR.

RT-PCR for assessing switching on of endogenous pluripotency genes and QRT-PCR for assessing the degree of switching off of transgene sequences, was carried out using primer sequences published by Takahashi et al. table S12 [45], except that in place of the published reverse primer (pMXs-AS3200 TTA TCG TCG ACC ACT GTG CTG CTG), we used our own designed primer, pMXs-AS3200v2 (TTA TCG TCG ACC ACT GTG CTG GCG) which had exactly the same sequence as the target pMXs vector backbone sequence and which therefore amplified more efficiently; also, for amplifying transgene mNanog, the forward primer GCT CCA TAA CTT CGG GGA GG was used. RNA was made using an RNeasy kit (Qiagen), then reverse transcription was carried out using a RetroScript kit (Ambion), using 2 ug template RNA in 20 ul reaction volume. 2 ul of 1∶10 dilution of cDNA product was used in a 25 ul qRT-PCR reaction. QRT-PCR was carried out on an Applied Biosystems StepOne Plus Real Time PCR machine, with StepOne software, using Applied Biosystems 2xSYBR green PCR mix+ROX and 60 degree Celsius anneal, Target gene transcript levels were compared to actin B control (actin B primers, Eurogentec), and subsequently to either fibroblasts harvested 5 days after infection with the reprogramming vectors (for transgene expression) or to undifferentiated HUES2, as appropriate [2].

### Assessment of Genome Integrity of hiPSC Lines

Genome integrity was analysed by running genomic DNA (extracted using Qiagen DNeasy Blood and Tissue kit, from samples at the same passage as for RNA above) on an Illumina Human CytoSNP-12v2.1 beadchip array (∼300,000 markers) and subsequent analysis using GenomeStudio and KaryoStudio software (Illumina), comparing the hiPSC lines to the parental normal human dermal fibroblasts (NHDF-OX1).

## Supporting Information

Figure S1 **PSC-MC harvested at day 356.** A) Phenotype of MC derived from the PSC line (HUES-2) after 356 days. Surface expression of CD14 and CD45 were measured by flow cytometry. Histograms represent surface staining (black line) compared to the isotype control (shaded gray).
(TIFF)

Figure S2 **PSC-MC and PSC-MDM production from multiple PSC lines.** A) Representative Forward Scatter (FSC) and Side Scatter (SSC) dot plot of harvested MC derived from the induced PSC line OX1-18 showing a gate around the homogenous cell population. B) Representative brightfield image of MDM derived from the induced-PSC line OX1-18 (scale bare 200 µM). C) Non-adherent MC derived from the PSC line (HUES-2) and induced-PSC lines (OX1-18, OX1-10 and OX1-23) were harvested from the supernatant of differentiation cultures and counted using a cell counter (Chemometec). The media were replaced for repeated PSC-MC harvests over a period of 13 week. Data represent the cumulative number of viable PSC-MC from 6 wells. D+E) Phenotype of MC (D) and MDM (E) derived from the PSC line (HUES-2) and induced-PSC lines (OX1-18, OX1-10 and OX1-23). Surface expression of CD14 and CD45 were measured by flow cytometry. Histograms represent surface staining (black line) compared to the isotype control (shaded gray).
(TIF)

Figure S3 **PSC-MC production and PSC-MDM characterisation using the fully defined protocol.** A) Non-adherent PSC-MC were harvested from the supernatant of differentiation cultures and counted using a cell counter (Chemometec). The media were replaced for repeated PSC-MC harvests over a period of 10 week. Data represent the cumulative number of viable PSC-MC from 6 wells. B) PSC-MDM were generated xenofree by culturing PSC-MC for 7 days in X-VIVO^TM^15 supplemented with M-CSF. Surface expression of CD14, CD16, CD163. MHC II and CD4 were measured by flow cytometry. Histograms represent surface staining (black line) compared to isotype control (shaded gray).
(TIF)

Figure S4 **PSC-MC are karyotypically normal.** KaryoStudio Detected Regions for monocytes derived from HUES2 and from iPS-OX1-19. Only chromosomes which contained regions detected by KaryoStudio as deviating from expected (indicated in ‘Found Region’ column by green for amplification, orange for deletion) are shown, as are the X and Y chromosomes (which, being single copy, are Called despite being the expected copy number). See Figure 9 for comparison and further explanation. The genes that these affect are listed in Table 1.
(TIF)

Figure S5 **PSC-MC production in the presence of Puromycin.** A) Puromycin selected PSC-MC (containing lentiviral vector expressing Puromycin resistance gene) or unselected PSC-MC were harvested from the supernatant of differentiation cultures and counted using a cell counter (Chemometec). The media were replaced for repeated PSC-MC harvests over a period of 8 week. Data represent the cumulative number of viable PSC-MC from 6 wells.
(TIF)

Methods S1 **Generation and culture of hiPSC lines.**
(DOCX)
