# Supplementary figures and images for: Efficient, Long Term Production of Monocyte-Derived Macrophages from Human Pluripotent Stem Cells under Partly-Defined and Fully-Defined Conditions
Source: PLoS One. 2013 Aug 12;8(8):e71098. doi: 10.1371/journal.pone.0071098 (PMC3741356; doi:10.1371/journal.pone.0071098)

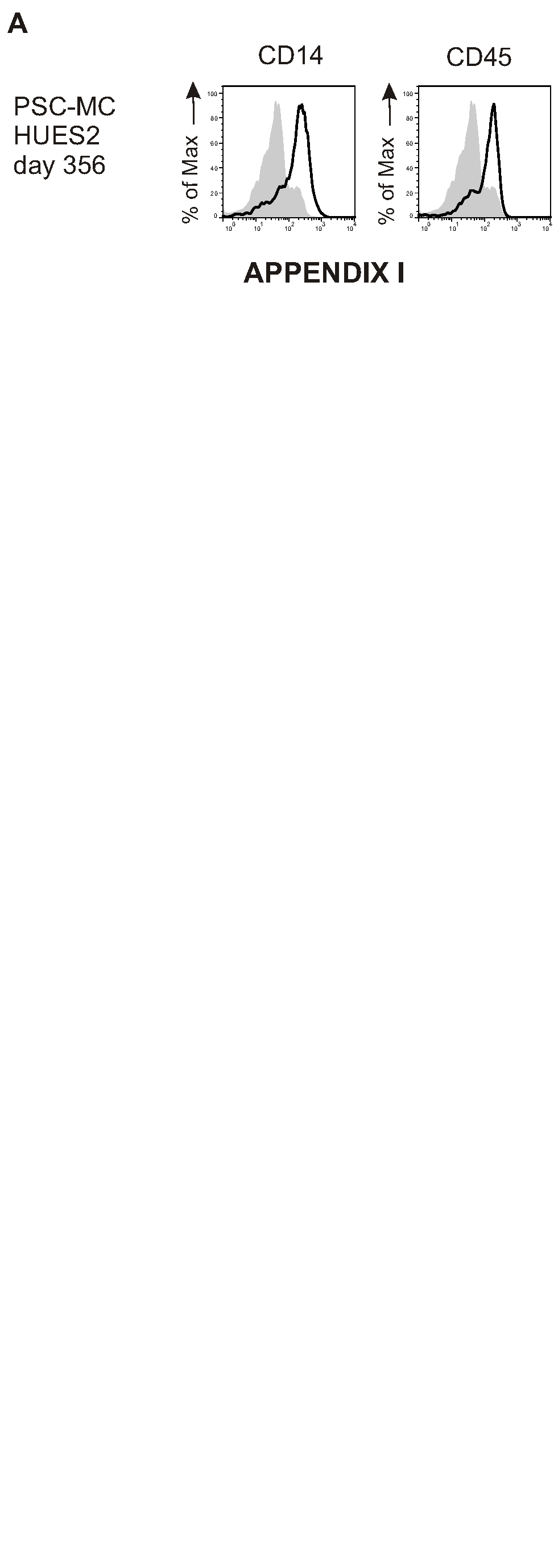

Supplement: Figure S1 — PSC-MC harvested at day 356. A) Phenotype of MC derived from the PSC line (HUES-2) after 356 days. Surface expression of CD14 and CD45 were measured by flow cytometry. Histograms represent surface staining (black line) compared to the isotype control (shaded gray). (TIFF) [file pone.0071098.s001.tiff]

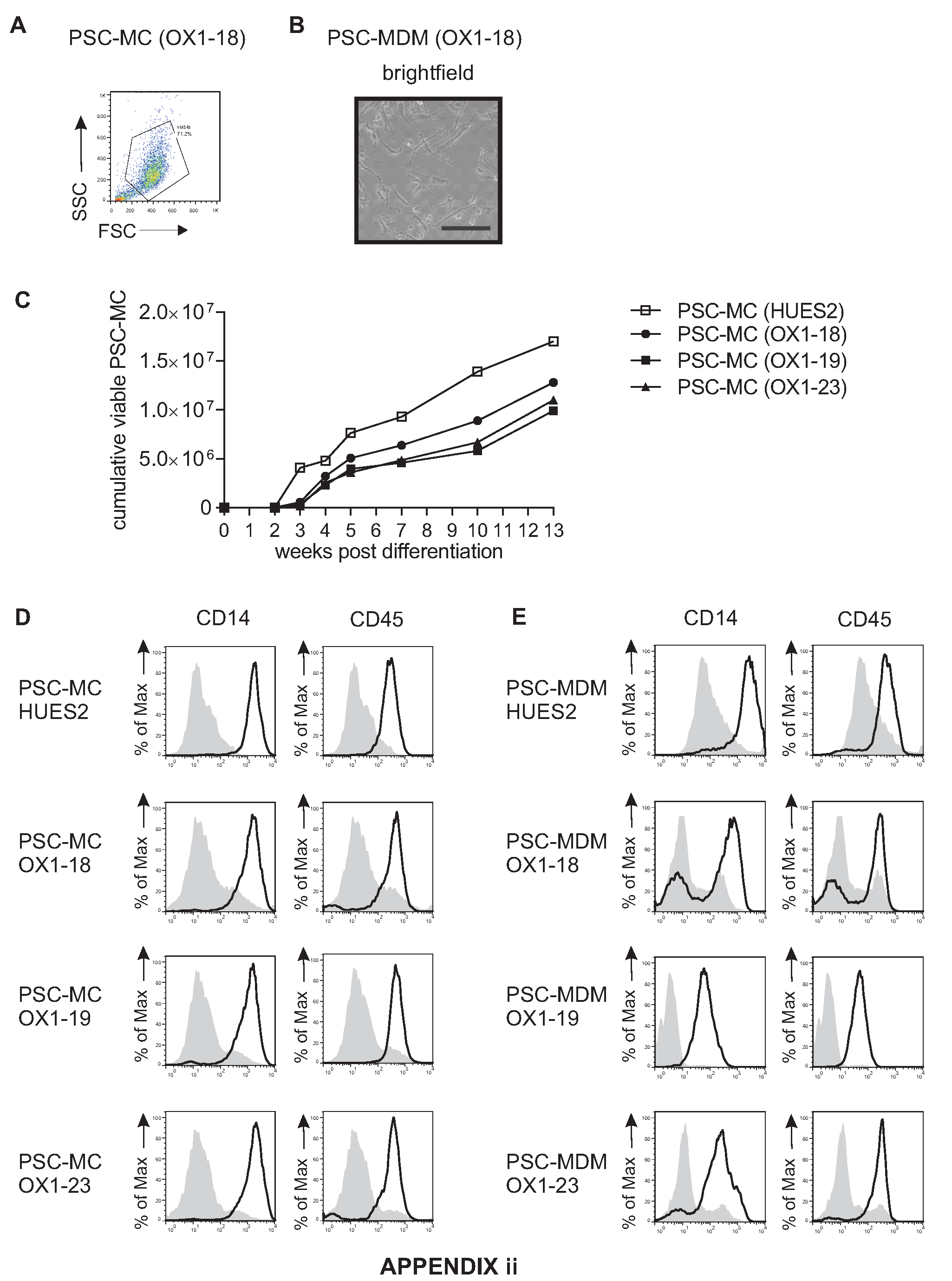

Supplement: Figure S2 — PSC-MC and PSC-MDM production from multiple PSC lines. A) Representative Forward Scatter (FSC) and Side Scatter (SSC) dot plot of harvested MC derived from the induced PSC line OX1-18 showing a gate around the homogenous cell population. B) Representative brightfield image of MDM derived from the induced-PSC line OX1-18 (scale bare 200 µM). C) Non-adherent MC derived from the PSC line (HUES-2) and induced-PSC lines (OX1-18, OX1-10 and OX1-23) were harvested from the supernatant of differentiation cultures and counted using a cell counter (Chemometec). The media were replaced for repeated PSC-MC harvests over a period of 13 week. Data represent the cumulative number of viable PSC-MC from 6 wells. D+E) Phenotype of MC (D) and MDM (E) derived from the PSC line (HUES-2) and induced-PSC lines (OX1-18, OX1-10 and OX1-23). Surface expression of CD14 and CD45 were measured by flow cytometry. Histograms represent surface staining (black line) compared to the isotype control (shaded gray). (TIF) [file pone.0071098.s002.tif]

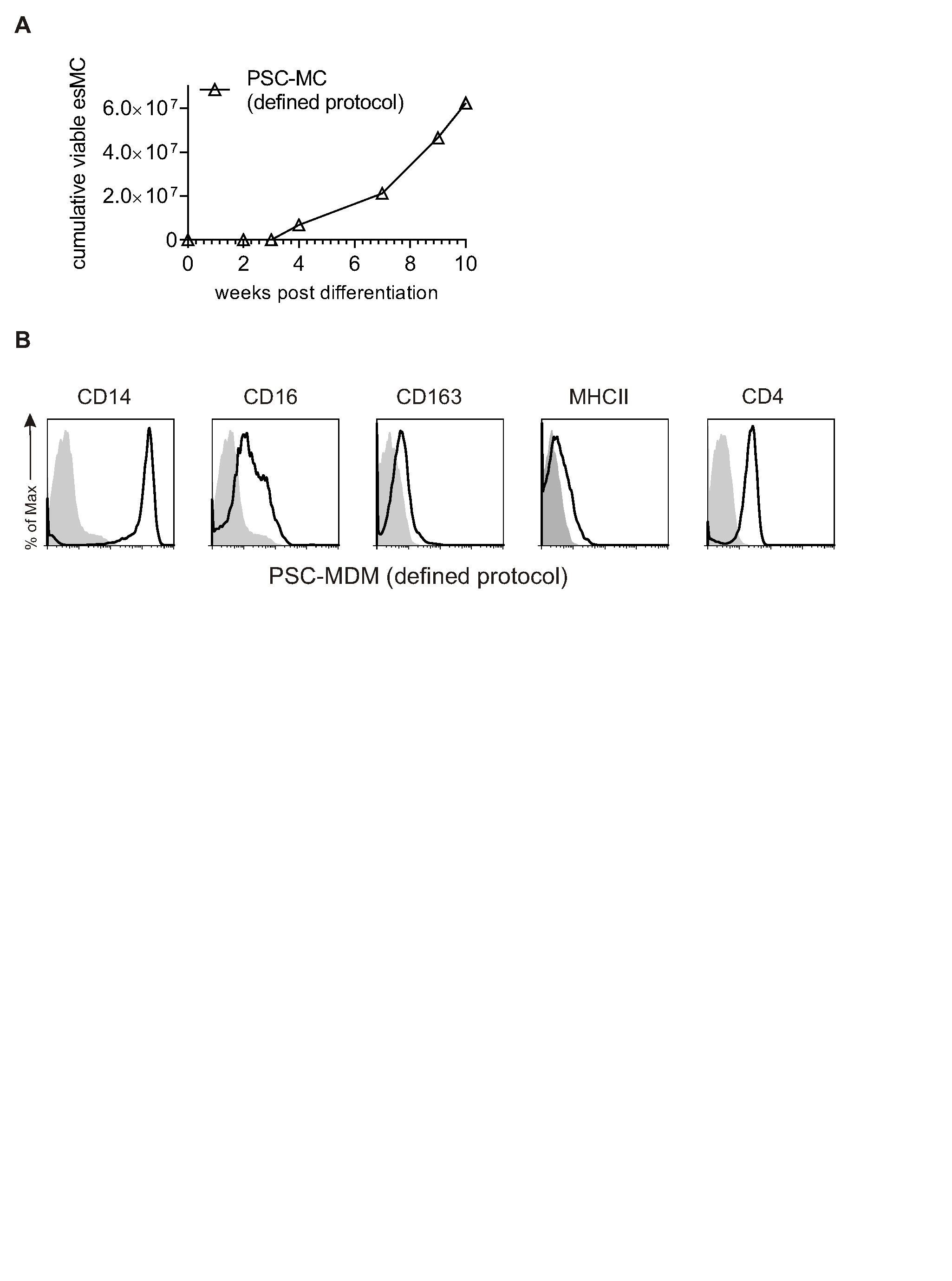

Supplement: Figure S3 — PSC-MC production and PSC-MDM characterisation using the fully defined protocol. A) Non-adherent PSC-MC were harvested from the supernatant of differentiation cultures and counted using a cell counter (Chemometec). The media were replaced for repeated PSC-MC harvests over a period of 10 week. Data represent the cumulative number of viable PSC-MC from 6 wells. B) PSC-MDM were generated xenofree by culturing PSC-MC for 7 days in X-VIVOTM15 supplemented with M-CSF. Surface expression of CD14, CD16, CD163. MHC II and CD4 were measured by flow cytometry. Histograms represent surface staining (black line) compared to isotype control (shaded gray). (TIF) [file pone.0071098.s003.tif]

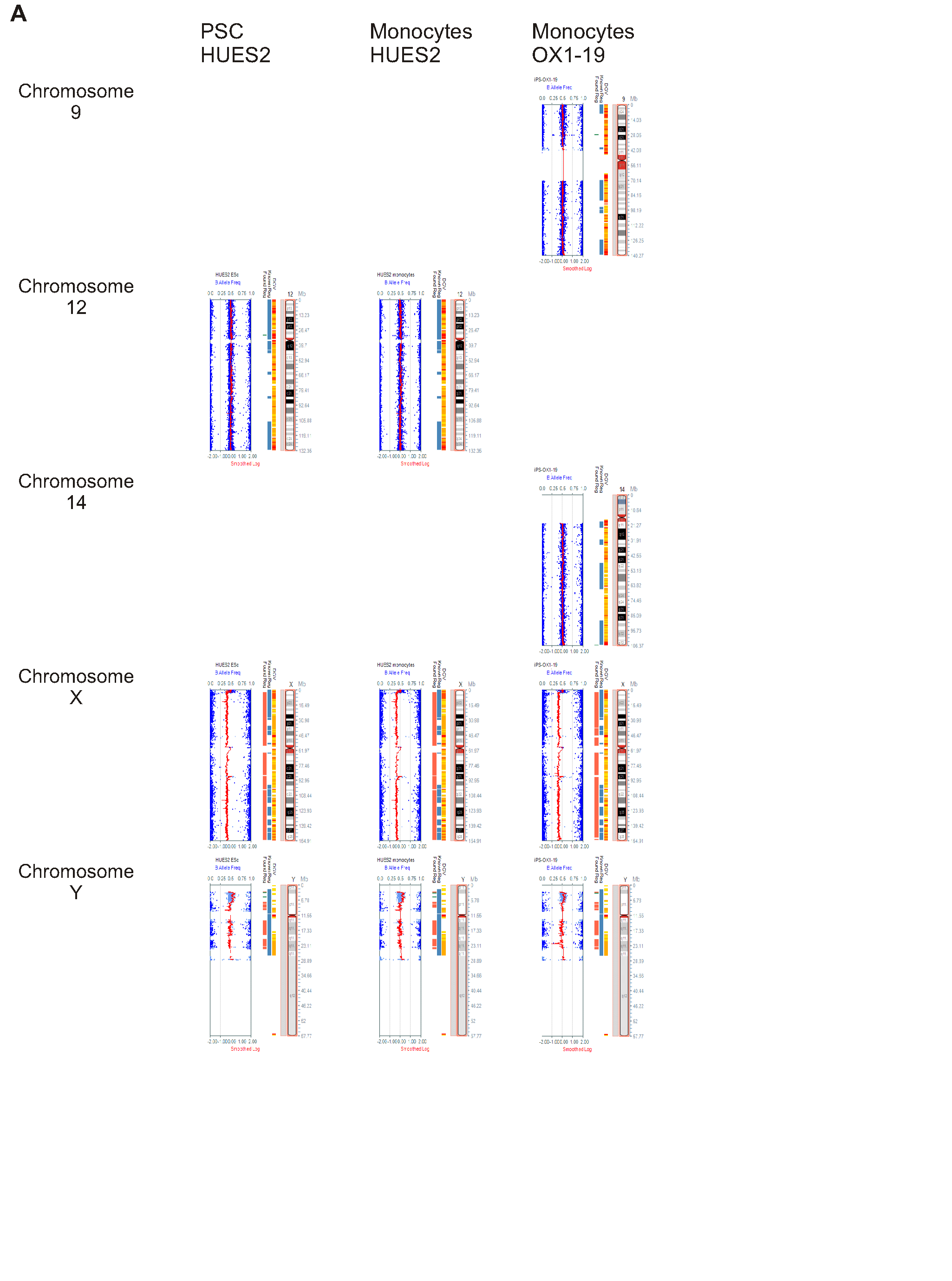

Supplement: Figure S4 — PSC-MC are karyotypically normal. KaryoStudio Detected Regions for monocytes derived from HUES2 and from iPS-OX1-19. Only chromosomes which contained regions detected by KaryoStudio as deviating from expected (indicated in ‘Found Region’ column by green for amplification, orange for deletion) are shown, as are the X and Y chromosomes (which, being single copy, are Called despite being the expected copy number). See Figure 9 for comparison and further explanation. The genes that these affect are listed in Table 1. (TIF) [file pone.0071098.s004.tif]

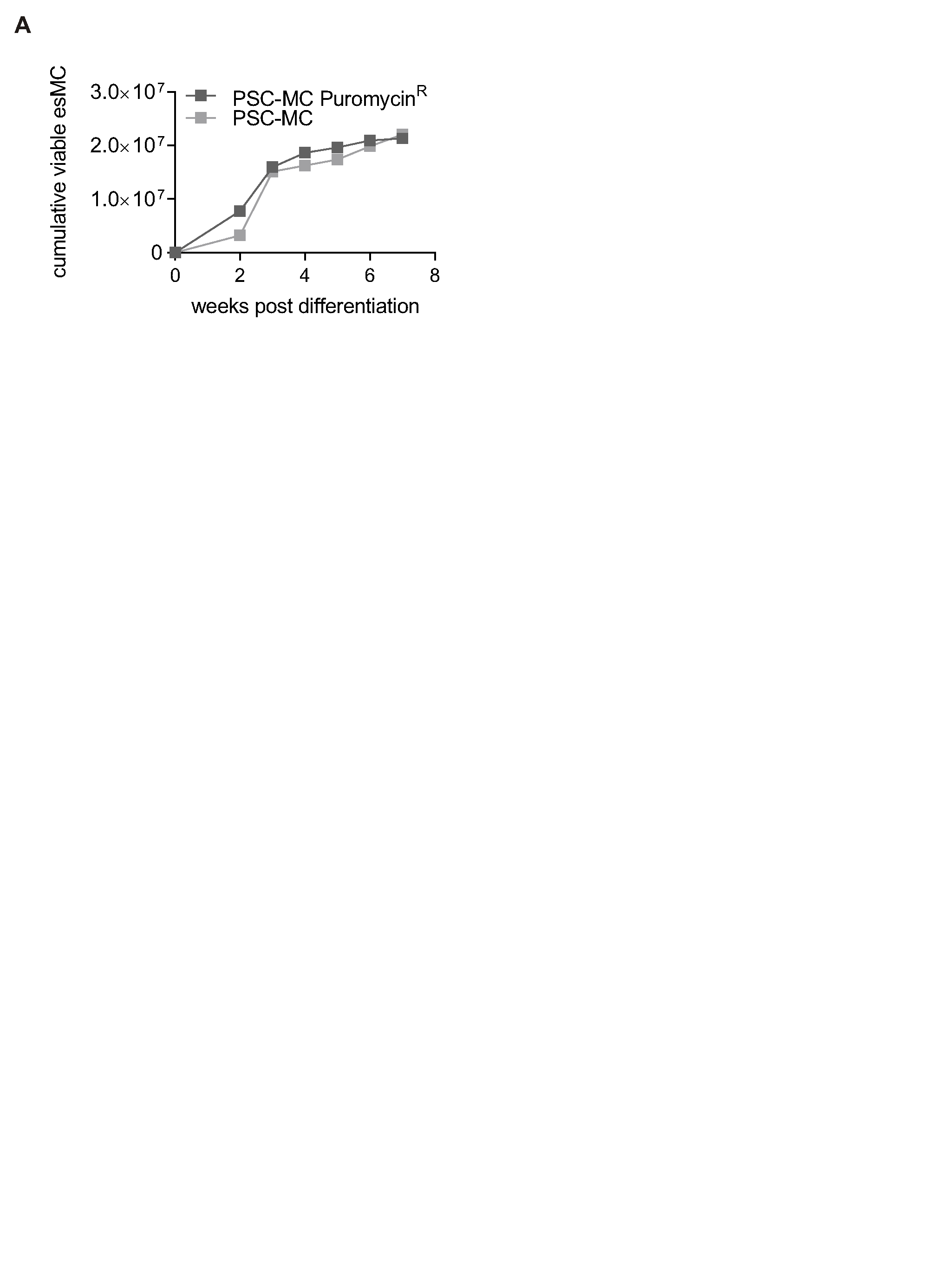

Supplement: Figure S5 — PSC-MC production in the presence of Puromycin. A) Puromycin selected PSC-MC (containing lentiviral vector expressing Puromycin resistance gene) or unselected PSC-MC were harvested from the supernatant of differentiation cultures and counted using a cell counter (Chemometec). The media were replaced for repeated PSC-MC harvests over a period of 8 week. Data represent the cumulative number of viable PSC-MC from 6 wells. (TIF) [file pone.0071098.s005.tif]
