# Supplementary material for: Efficient, Long Term Production of Monocyte-Derived Macrophages from Human Pluripotent Stem Cells under Partly-Defined and Fully-Defined Conditions
Source: PLoS One. 2013 Aug 12;8(8):e71098. doi: 10.1371/journal.pone.0071098 (PMC3741356; doi:10.1371/journal.pone.0071098)
Supplement: Methods S1 — Generation and culture of hiPSC lines. (DOCX) [file pone.0071098.s006.docx]

# Supporting Information

**Methods S1**

**Generation and culture of hiPSC lines**

iPS-OX1-18, iPS-OX1-19 and iPS-OX1-23 were all derived in-house from fibroblasts from a skin biopsy from a normal male donor, age 36 (see Ethics statements for use of adult blood and stem cell lines in Materials and Methods, main manuscript). pMXs plasmids encoding the reprogramming genes were obtained from Addgene (17220: pMXs-hc-MYC, 17219: pMXs-hKLF4, 17218: pMXs-hSOX2, 17217: pMXs-hOCT3/4, 13354: pMXs-mNanog) and were as used in the original paper by Takahashi and Yamanaka [[1](#_ENREF_1)]. Nanog was included to improve reprogramming efficiency, as the lines were part of a larger study that included aged donors. The plasmids were packaged using the Plat-GP retroviral packaging cell line (containing retroviral gag and pol genes) and cotransfected with VSV-G, using PEI, to make iPS retrovirus vectors. Reprogramming was carried out following [[1](#_ENREF_1)]; 50,000 Fibroblasts were infected on days 0 and 1with an equal volume of each virus supernatant, plus 5µg/ml polybrene and spinoculation (1200g for 45 minutes at 16 ^o^ C). They were transferred onto ‘Pathology Oxford’ outbred mitomycin C-inactivated mouse embryonic feeder cells (MEF) on 0.1% gelatin coated plates (Sigma) on day 4, and from day 5 onwards were cultured in standard KnockOut serum replacement medium (Life Technologies) supplemented with 50 µg/ml ascorbic acid and 0.5 µM Valproic acid (both from Sigma), replacing 50% medium on alternate days, and substituting with MEF-conditioned medium from day 10 onwards. Colonies were picked and lines established. Minimal characterisation of the lines was initially carried out [[2](#_ENREF_2)]; here more detailed analyses have been conducted. For RNA and DNA analyses hiPSC lines were adapted to feeder-free culture conditions by three passages of enzymatic dissociation (TryplE Express, Gibco) onto Matrigel coated plates (BD Matrigel hESC-qualified Matrix) in mTeSR™1 (StemCell Technologies) supplemented with Rock inhibitor Y27632 (10μM; Calbiochem) on the day of passage. The number of feeder-free passages was always kept low (<10) to reduce the likelihood of genetic change.

**Flow Cytometry**

For analysis of cell surface molecules, 0.5-1 x 10^6^ cells were washed and stained in flow cytometry buffer consisting of PBS, human IgG (10 µg/mL Sigma), FCS (1 % Hyclone) and sodium azide (0.01 %), with an antibody or an isotype-matched control (with same fluorophore, from the same manufacturer) on ice for 30 min. For two-colour staining, two antibodies or two isotype controls (attached to different fluorophores) were added together. Cells were fixed with 4 % paraformaldehyde in PBS. Fluorescence was measured using a FACS Calibur (Becton Dickinson), and data was analysed using FlowJo software on marked cell populations on FSC-SSC dot plots.

The following anti-bodies (clone, isotype controls, supplier) were used: CD14-APC (MEM-15, IgG1-APC, Immunotools), CD45 (MEM-28, IgG1-APC, Immunotools), CD16 (LNK1, IgG1-APC, Immunotools), CD163-PE (GHI/61, IgG1ĸ-PE, BD), MHCII CD4-APC (11830, IgG2a-APC, R&D Systems),

# Supporting References

1. Takahashi K, Okita K, Nakagawa M, Yamanaka S (2007) Induction of pluripotent stem cells from fibroblast cultures. Nature protocols 2: 3081-3089.

2. Cowley SA, Karlsson KR, van Wilgenburg B, Carter G, Browne C, et al. (2012) Macrophages from human Pluripotent Stem Cells. In: Takahashi R, Kai H, editors. Handbook of Macrophages: Life Cycle, Functions and Diseases: Nova Science Publishers. pp. 83-120.
